# A citation analysis and scoping systematic review of the operationalization of the Practical, Robust Implementation and Sustainability Model (PRISM)

**DOI:** 10.1186/s13012-022-01234-3

**Published:** 2022-09-24

**Authors:** Borsika A. Rabin, Julie Cakici, Caitlin A. Golden, Paul A. Estabrooks, Russell E. Glasgow, Bridget Gaglio

**Affiliations:** 1 Herbert Wertheim School of Public Health and Human Longevity Science, University of California San Diego, 9500 Gillman Drive, La Jolla, CA 92037 USA; 2 UC San Diego Altman Clinical and Translational Research Center Dissemination and Implementation Science Center, UC San Diego, 9500 Gillman Drive, La Jolla, CA 92037 USA; 3 Dissemination and Implementation Science Program, Adult & Child Center for Health Outcomes Research & Delivery Science; and Department of Family Medicine, University of Colorado, 1635 Aurora Ct, Aurora, CO 80045 USA; 4 School of Public Health, San Diego State University, 5500 Campanile Drive, San Diego, CA 92182 USA; 5 University of Nebraska Medical Center, 42nd and Emile St, Omaha, NE 68198 USA; 6 Health & Kinesiology, College of Health, University of Utah, 248 HPER North, 260 South 1850 East, Salt Lake City, UT 84112 USA; 7 Patient-Centered Research, Evidera, 7101 Wisconsin Ave, Suite 1400, Bethesda, MD 20814 USA

**Keywords:** PRISM, Pragmatic, Review, Framework, Context, RE-AIM, Implementation, Sustainability

## Abstract

**Background:**

The Practical, Robust Implementation and Sustainability Model (PRISM) was developed in 2008 as a contextually expanded version of the broadly used Reach, Adoption, Effectiveness, Implementation, and Maintenance (RE-AIM) framework. PRISM provides researchers a pragmatic and intuitive model to improve translation of research interventions into clinical and community practice. Since 2008, the use of PRISM increased across diverse topics, populations, and settings. This citation analysis and scoping systematic review aimed to assess the use of the PRISM framework and to make recommendations for future research.

**Methods:**

A literature search was conducted using three databases (PubMed, Web of Science, Scopus) for the period of 2008 and September 2020. After exclusion, reverse citation searches and invitations to experts in the field were used to identify and obtain recommendations for additional articles not identified in the original search. Studies that integrated PRISM into their study design were selected for full abstraction. Unique research studies were abstracted for information on study characteristics (e.g., setting/population, design), PRISM contextual domains, and RE-AIM outcomes.

**Results:**

A total of 180 articles were identified to include PRISM to some degree. Thirty-two articles representing 23 unique studies integrated PRISM within their study design. Study characteristics varied widely and included studies conducted in diverse contexts, but predominately in high-income countries and in clinical out-patient settings. With regards to use, 19 used PRISM for evaluation, 10 for planning/development, 10 for implementation, four for sustainment, and one for dissemination. There was substantial variation across studies in how and to what degree PRISM contextual domains and RE-AIM outcomes were operationalized and connected. Only two studies directly connected individual PRISM context domains with RE-AIM outcomes, and another four included RE-AIM outcomes without direct connection to PRISM domains.

**Conclusions:**

This is the first systematic review of the use of PRISM in various contexts. While there were low levels of ‘integrated’ use of PRISM and few reports on linkage to RE-AIM outcomes, most studies included important context domains of implementation and sustainability infrastructure and external environment. Recommendations are provided for more consistent and comprehensive use of and reporting on PRISM to inform both research and practice on contextual factors in implementation.

**Supplementary Information:**

The online version contains supplementary material available at 10.1186/s13012-022-01234-3.

Contributions to the literature

- We provide an overview of the use of PRISM for planning, implementation, or evaluation. Key characteristics for PRISM use include study location, consideration of health equity, target population, setting, health topics, study designs, methods, level of analysis, and operationalization of PRISM context domains and RE-AIM outcomes.
- We illustrate how PRISM has been used in across a wide range of applications and which PRISM domains have been reported most and least often.
- Recommendations are provided for researchers and practitioners for the more consistent and comprehensive use of and reporting on PRISM to inform the consideration of contextual factors in implementation.

## Introduction

The field of implementation science has made significant advancements towards understanding how evidence-based practices, programs, and policies are adopted and implemented in different clinical and community settings [1, 2]. While there is a wide range of evidence-based interventions, it is still the case that less is known about successfully implementing and sustaining these programs in a range of settings [3, 4]. Implementation science has emphasized the development and use of theories, models, and frameworks (TMF) to guide and understand translation of research into practice [5, 6] and has prioritized understanding how health care and public health programs interact with context and both implementers and beneficiaries to influence adoption, engagement, equity [7], implementation, reach, effectiveness, and sustainment [8–11].

There is increasing interest [12, 13] but still limited understanding of which contextual factors [3, 14–17] have an impact on the initial uptake; equitable implementation, reach, effectiveness; and sustained use of complex health interventions [18] in a variety of clinical and community settings, which inhibits the translation of research into practice [10]. More specifically, there is a need to document and understand the impact of the dynamic context in which interventions are integrated [19–23]. The Practical, Robust Implementation and Sustainability Model (PRISM) was developed to fill this need using key concepts from research on and models of chronic care, the diffusion of innovations, quality improvement, and measures of population-based effectiveness for translating research into practice [3, 4].

**Fig. 1:**
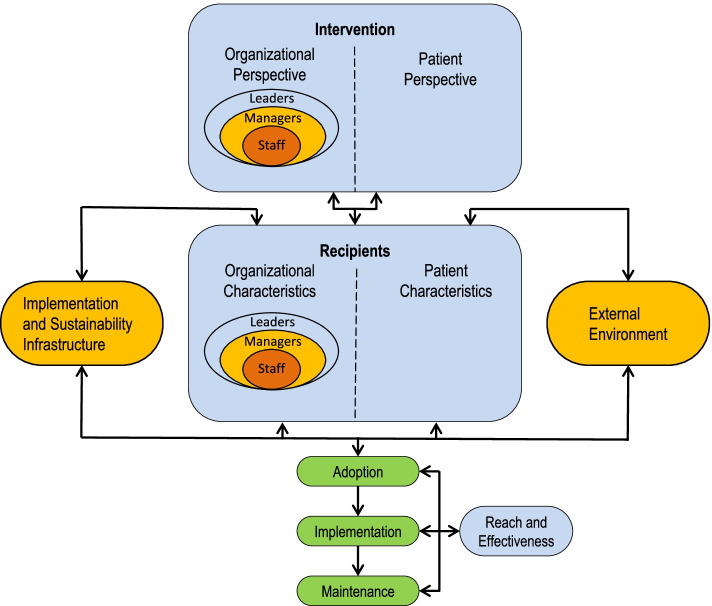
The Practical, Robust Implementation and Sustainability Model

PRISM was developed as a contextually expanded version of the broadly used Reach, Adoption, Effectiveness, Implementation, and Maintenance (RE-AIM) framework, and as a pragmatic and intuitive model to improve translation of research-tested interventions into clinical and community practice and ultimately population health impact [24]. PRISM can be considered a determinant and evaluation framework in the classification suggested by Nilsen [25] and, as illustrated in Fig. 1, considers how perspectives of the program, policy, or intervention design; the external environment; the implementation and sustainability infrastructure; and the characteristics of multiple levels of “recipients” (e.g., implementers, beneficiaries) influence program adoption, implementation, and maintenance. Within the program or intervention design domain and the recipient domain, PRISM incorporates the perspectives of both the patients (recipients or participants) and the organizational members at different levels of influence (e.g., top leadership, mid-level managers, and frontline staff) to help understand what factors within and external to the organization need to be considered and addressed for successful implementation and sustainability of complex interventions [26]. A relatively unique aspect of PRISM, compared to most other models addressing context, is explicit inclusion of the domain of the “implementation and sustainability infrastructure” (e.g., clear roles and responsibilities related to this program; timely data reporting capabilities, strong communication channels). Inclusion of this domain was based on experience in healthcare settings in which those settings that were able to implement and sustain programs most consistently had the type of infrastructure and support resources noted in this domain. The outcome measures hypothesized to be influenced by PRISM contextual factors include RE-AIM outcomes of reach, effectiveness, adoption, implementation, and individual- and organizational-level maintenance (www.re-aim.org) [4, 27].

Since 2008, PRISM has been used across diverse topics, populations, and settings. The purpose of this citation analysis and scoping systematic review was to explore and describe how PRISM has been used, how it has been operationalized, for what populations and settings, and with what outcomes. Specifically, the review aimed to (1) summarize use of PRISM to date; (2) identify and address conceptual and methodological issues to potentially improve research in this area; and (3) provide recommendations for research teams and practitioners about using PRISM to improve translation of evidence-based interventions in a variety of clinical and community settings.

## Methods

We conducted a systematic scoping literature review to determine how PRISM has been used. A literature search was conducted using three databases PubMed, Web of Science, and Scopus. The search algorithm included ((“Practical” AND “Robust” AND “Implementation” AND “Sustainability” AND “Model”) OR (“Practical, Robust Implementation and Sustainability Model”)) OR (“Practical Robust Implementation and Sustainability Model”).

The date range was from 2008 (publication date of the original PRISM manuscript) through September 2020. Further, following the approach described by Bergstörm et al. [28], we conducted a reverse citation search of the original PRISM article (i.e., index article) in PubMed reviewing all papers that cited this index paper to identify studies that used PRISM [4]. Only peer-reviewed articles were included in this review. First, duplicates were removed from the results. Full-text articles were obtained for all articles identified in the searches and were independently reviewed by two researchers (BR, JC) to determine eligibility. Articles were excluded if they did not cite or specify PRISM or were not yet published in a peer-reviewed journal. We adapted and refined categories previously developed by Field et al. [29] to classify the extent PRISM was used in the articles (i.e., referenced the model, framing a field or in a review, informed by the model, directed by the model, or integrated the model into the study design) (Table 1). Classification of articles per the Field et al categories [29] was done by two members of the team (BR, JC). Discrepancies in classification were resolved through consensus discussion and the involvement of the other members of the abstraction team (CG, BG). Because one purpose of this review was to explore the degree to which PRISM propositions were supported (i.e., relationships between PRISM contextual factors and RE-AIM outcomes), only studies that met the criteria for ‘integrated use,’ which required explicit linking of study activities to PRISM domains and/or subdomains, of the PRISM framework were included [29]. Following the database searches and initial eligibility screening, the list of included articles was circulated to an expert panel of implementation science researchers via a national listserv to obtain recommendations for additional articles that described an integrated use of PRISM not identified in the original search.

**Table 1:** Categories and definitions for the use of the Practical, Robust Implementation and Sustainability Model (PRISM) in published peer-reviewed literature, adapted from Field et al. (2014) [29]

**Framing Field/Area**	The PRISM framework is used to understand the current state of the field and guide directions for future research and practice.
**Review**	The PRISM framework has been described in a review of frameworks as one of the frameworks.
**Referenced**	The PRISM framework is cited (often not named) to establish a broader context for the Implementation Science field with no further explanation of direct relevance to this study.
**Informed**	The PRISM framework cited and named often in the introduction or discussion as it relates to this study or future related studies but no explanation of direct use.
**Directed**	The PRISM framework had influenced project planning or design but no specifics or examples are given.
**Integrated**	The PRISM framework was integral to the planning, design, delivery, evaluation, interpretation, and/or dissemination and scale-up and scale-out of the study/project.
**Exclude**	The PRISM framework was not cited in the paper.

All articles that were categorized as integrating PRISM into their study design (i.e., integrated use [29]) were selected for full abstraction. Articles that described the same study were bundled as primary and companion papers and were abstracted together. Data abstraction was conducted by four researchers (BR, BG, CG, and JC). Data abstraction forms were developed based on prior work of the co-authors in reviewing the use of the RE-AIM framework [30, 31]. We expanded these abstractions forms to include PRISM contextual domains of characteristics of the intervention, characteristics of the recipients (including the multi-level organizational setting), implementation and sustainability infrastructure, and external environment. The abstraction form was then iteratively refined as the team tested it on the abstraction of four studies and then programmed into a REDCap database [32, 33]. The rest of the eligible articles were assigned to pairs of researchers to independently abstract. The pairs of coders met to review their abstractions, resolve discrepancies, and reach consensus. Consensus, rotation of abstraction teams, and iterative group discussions were used to ensure the accuracy and consistency of data abstraction.

The database was used to capture information from three key domains: study characteristics (setting, design, population, etc.), PRISM contextual domains, and RE-AIM outcomes [32, 33]. Articles were categorized as addressing planning/development, dissemination, implementation, evaluation, and/or sustainment. To describe the degree (i.e., quality and extent) of operationalization of PRISM domains, we used a scale of 0-5 in which 0 indicated poor operationalization and 5 indicated excellent operationalization. Ratings were separately made for each type of use of PRISM (i.e., planning/development, dissemination, implementation, evaluation, and/or sustainment). Two abstractors per article/study rated the degree of operationalization and used a consensus approach to decide on the final rating. They also documented the rational for their rating in a qualitative manner. If articles included lessons learned about operationalizing PRISM, this information was also systematically abstracted (see Additional file 1). Key themes from lessons learned were synthesized based on the review of this data by the lead author.

## Results

### Included studies

**Fig. 2:**
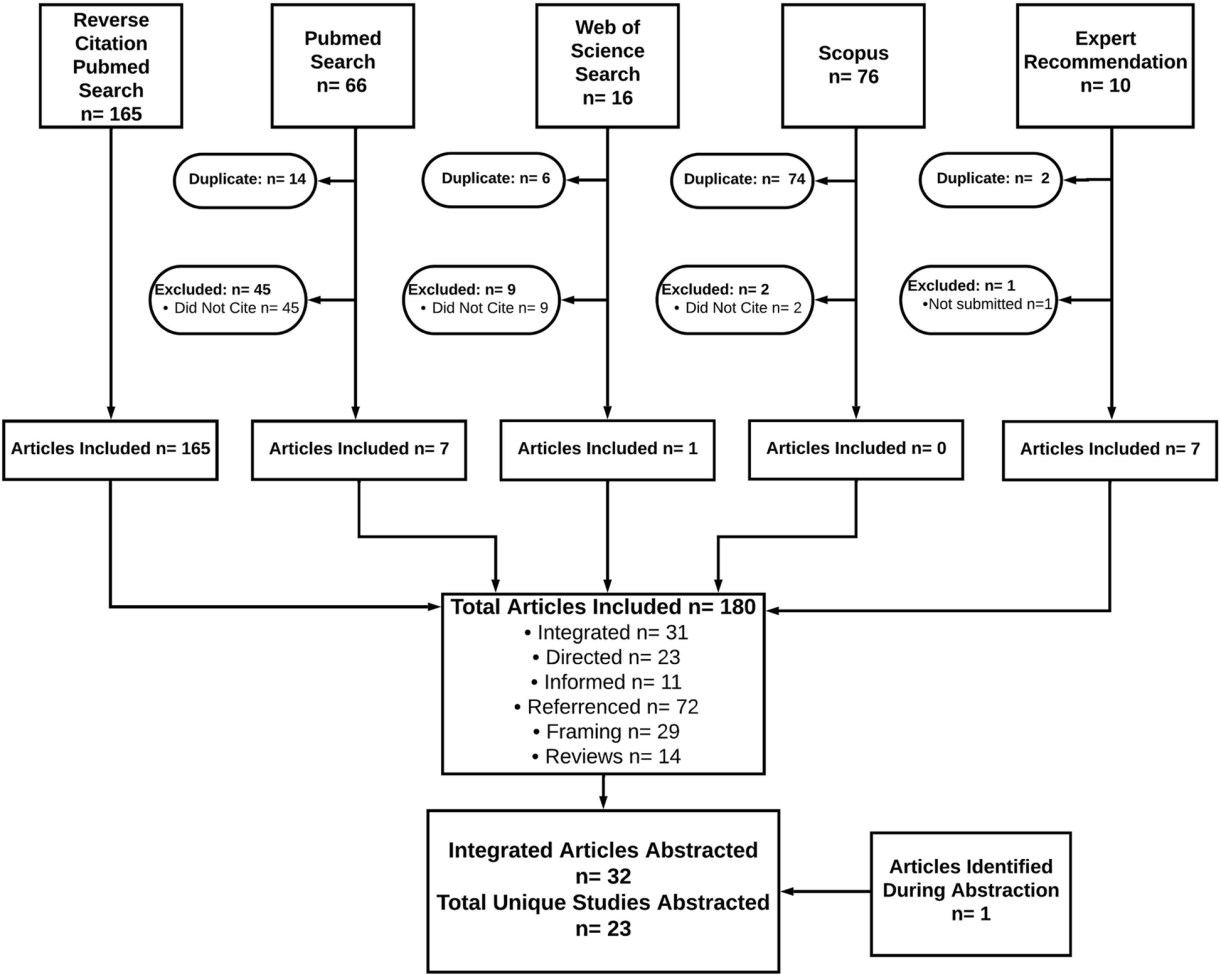
Preferred Reporting Items for Systematic Reviews and Meta-Analyses (PRISMA) diagram of systematic review of articles using Practical, Robust Implementation and Sustainability Model (PRISM)

**Fig. 3:**
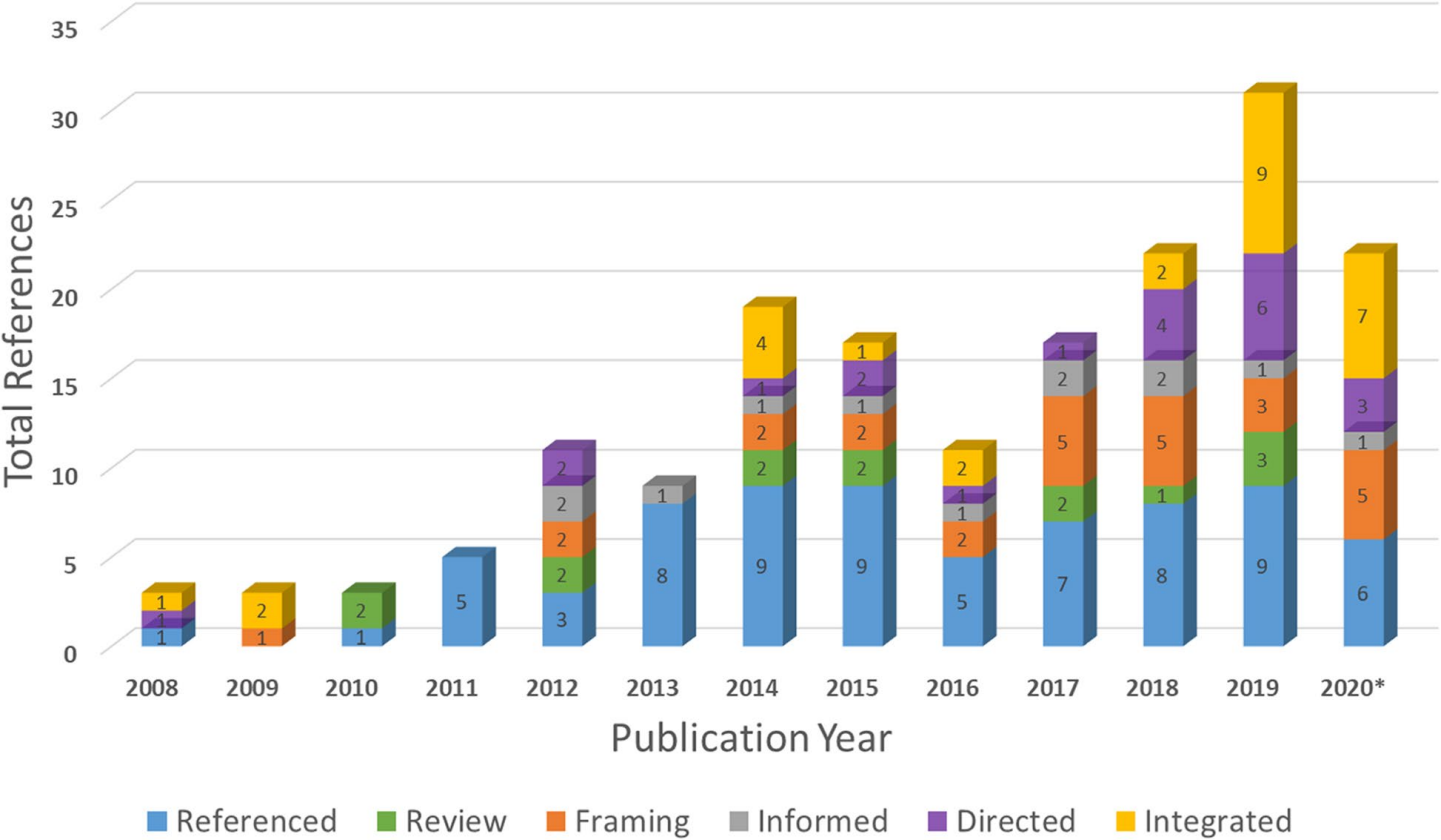
Number of articles using the Practical, Robust Implementation and Sustainability Model (PRISM) across years (2008 through September 2020*)

Of the 237 unique articles identified, 57 were excluded for not meeting the initial inclusion criteria (Fig. 2 and Table 1). A total of 180 articles were identified as using PRISM to some degree (i.e., referenced the model, framing a field or in a review, informed by the model, directed by the model, or integrated the model into the study design). As shown in Fig. 3, the number of publications increased over time as we moved from 2008 (*n*=3) to 2019 (*n*=31). Overall increases in the proportion of directed and integrated articles were also observed (see Table 1 for definitions).

Of the 180 initially identified articles, 29 (16.1%) included PRISM as a potential means for understanding the current state of research or advancing research in a specific field (i.e., framing), and 14 articles (7.8%) included PRISM in a review of frameworks (i.e., reviews). Eleven articles (6.1%) mentioned that PRISM informed their study without specifying how the model was used (i.e., informed), and another 23 articles (12.8%) provided more details as to how PRISM was used in developing their study but lacked specific details about how the domains were operationalized (i.e., directed). Seventy-two articles (40.0%) referenced PRISM in either the introduction or discussion as an alternative model that was not used in their study methods (i.e., referenced). Finally, 31 articles (17.2%), representing 23 unique studies, were determined to integrate PRISM into their study design and were abstracted (Fig. 2) [29]. One additional article was added to the final abstraction as it was identified as a later published result for an initially included protocol article (the total number of publications in the integrated category was *n*=32). For the full list of included papers, their categorization by use, and search source, see Additional file 2.

### Study characteristics

Of the 23 abstracted studies, 17 were conducted in the USA, and only three were conducted in low-middle income countries. Thirteen studies addressed primary or secondary prevention as the main topic area followed by seven studies related to mental health, four on veterans’ health, three on cancer, two on infectious disease, two on reproductive health (including OBGYN), one on clinical guidelines, and eight were coded as other topics. Eighteen of the studies addressed health equity, as defined as “reducing and ultimately eliminating disparities in health and its determinants that adversely affect excluded or marginalized groups” [34] (Table 2). Most frequent operationalization of addressing health equity was through the focus of the study on underserved populations.

**Table 2:** Summary of original studies included in a systematic review of the use of the Practical, Robust Implementation and Sustainability Model (PRISM)

Citation	Study location (health disparity Y/N)	Intervention description	Implementation strategy	Lessons learned	Study conclusions/findings
**Ameling, J. M., et al. (2014) **[35]	USA (Y- Underserved)	Patient-centered behavioral self-management interventions to improve hypertension control among urban African Americans receiving primary care	N/R	N/R	Use of hybrid methodologies to adapt interventions for populations experiencing health disparities to improve interventions’ translation to real clinical practice settings and enhance interventions’ sustained effectiveness. Focused on PRISM construct of multiple levels of recipients—organizational and patient characteristics and perceptions of the intervention. Their approach resulted in numerous potential participant-driven intervention modifications to improve the potential effectiveness of hypertension self-management interventions for urban African Americans. Investigators planning similar approaches should consider the substantial resources that similar efforts may require to obtain generalizable perspectives. Stakeholders’ input revolved around their: perceived potential for interventions to improve clinical practice, desired features of interventions, suggested ways to enhance interventions’ cultural relevance, and threats to interventions’ sustained effectiveness.
**Ayele, R. A., et al. (2017)** [36] (Ayele, R. A., et al. (2019) [37]; McCreight, M. S., et al. (2019) [38])	USA (Y- Veterans)	Transition of care quality improvement	Audit and Feedback	N/R	Four major themes emerged where participants consistently discussed that transitions were delayed when they were not able to (1) identify patients as veterans and notify VA primary care of discharge, (2) transfer non-VA hospital medical records to VA primary care, (3) obtain follow-up care appointments with VA primary care, and (4) write VA formulary medications for veterans that they could fill at VA pharmacies. Each theme was attributed in part to external environment and implementation and sustainability infrastructure contextual factors. The initial theme also was attributed in part to recipient (patient-level) characteristics. Participants also discussed factors involved in smooth transition and recommendations to improve care coordination suggesting positive implementation and sustainability infrastructure context factors also existed.
**Ekawati, F.M., et al. (2019) **[39] (Ekawati, F.M., et al. (2020) [40])	Indonesia (Y- LMI Country)	Hypertension Disorders in Pregnancy management pathways contextualized to Indonesian primary care settings (to be developed)	N/R	N/R	Even though Indonesian antenatal care and referrals are generally accessible, there are many challenges and fragmentation of HDP management. The most prominent challenge was related to recipient characteristics—the primary care providers’ lack of confidence in performing the management and a program/intervention factor related to the urgent need of practice guidelines in primary care that had not been appropriately described in the literature. Further development of an evidence-based primary care-focused guidance will potentially improve recipient characteristics—primary care providers’ skills to perform optimal HDP management and provide appropriate education to their patients. Protocol paper.
**Esses, S. A., et al. (2019) **[41]	USA (N)	Three educational strategies/ interventions to educate families about Post-Intensive Care Syndrome (PICS) symptom recognition and management	N/R	N/R	The survey indicated that all 3 interventions minimally disrupted workflow and all were recognized as useful. To ensure sustainable implementation, the characteristics of the unit should be considered when selecting an educational program. When parents/caregivers of pediatric ICU patients are given targeted education about PICS, their knowledge of the syndrome, its signs and symptoms, how to contact a social worker, self-management techniques, and available resources increases. In general, education about PICS itself, and how a family might receive assistance during their child’s ICU stay, is well supported by PICU nurses and perceived to be both important and not significantly disruptive to daily workflow. Overall, the costs of such a program are relatively low. Therefore, it is feasible for significant PICS-related education and family support to be provided in almost any PICU setting in a locally sustainable fashion.
**Gopalan, G, et al. (2016) **[42] (Gopalan, G., et al. (2019) [43]; Hooley, C., et al. (2020) [44])	USA (Y- Underserved)	4 Rs and 2 Ss for Strengthening Families Program: Caregiver engagement and behavioral parent training and family therapy strategies to support family-level influences on disruptive behavior disorders	Task-shifting Program Adaptations	PRISM is utilized in the current study as a framework for intervention adaptation into a new setting such that the resulting modified 4R2S intervention is maximized for subsequent implementation success. Use of an implementation framework may be increasingly necessary to guide basic intervention development to increase the likelihood that newly developed interventions are integrated into everyday practice.	Advisory board members reported difficulties engaging families, heavy workloads, and conflicting implementation initiatives. While 4R2S was perceived as generally aligned with their organization’s mission, modifications to the intervention and to agency procedures were recommended to promote implementation success. Suggested modifications to the existing 4R2S training and supervision are discussed. Findings underscore the importance of understanding the experiences of CW service providers, which can inform future efforts to implement child mental health EBIs in CW services. (Gopalan, G., et al. [43]). Recommendations included adjusting curriculum to better fit the culture of recipients and conveying the importance of openness and respect to providers (Hooley, C., et al. [44]).
**Gopalan, G, et al. (2014)** [45]	USA (Y- Underserved)	4 Rs and 2 Ss for Strengthening Families Program: Caregiver engagement and behavioral parent training and family therapy strategies to support family-level influences on disruptive behavior disorders	Adaptation based on Collaborative Approach Training Technical Assistance Skill Development	The PRISM model could further serve as a guide in the development phase of evidence-informed practices to pre-empt many of common agency, provider, and consumer level challenges of implementation. PRISM and a collaborative approach guided the revision of the 4Rs 2 Ss program to increase agency-level uptake of the program.	Facilitators and directors reported an overall positive experience in implementing the 4 Rs Program. Given that every clinic functions somewhat differently, flexibility in the implementation of the 4 Rs Program is vital. To advance the first statewide implementation of the 4 Rs Program, CTAC staff utilized PRISM to guide model modifications serving to enhance agency-level uptake. A collaborative approach guided model revisions such that the resulting program manual was co-created by family members, providers, and research staff to promote the existing evidence base in “user friendly” language. While the main components of the model based on the evidence-base remain the same, the revised version is streamlined to cut down on complexity, reduce the number of materials, better fit into current clinic structures, and is supported through a learning collaborative process.
**Henderson, V., et al. (2020)** [46]	USA (Y- Underserved)	Mile Square Accessible Mammogram Outreach and Engagement (Mi-MAMO): Breast cancer screening and navigation program	Patient Navigators Network Weaving	An integrated framework using the PRISM implementation science framework, grounded in a socioecological approach, is a feasible model to implement standard-of-care breast cancer screening.	Between January and December 2017, 103 women received a screening mammogram at Mile Square Health Centers. To increase screening rates, Mi-MAMO was started in August 2017. Between January and December 2018, the number of women who received a screening mammogram increased to 567. Twenty-four percent (*n* = 185) completed diagnostic services, and 10 women received positive breast cancer diagnoses (mean age, 49.7 years); all successfully navigated to treatment. Deploying an integrated framework for patient navigation programs can increase breast cancer screening utilization and awareness among under-resourced populations at higher risk for breast cancer.
**Knudsen, HK., et al. (2020)** [47]	USA (Y- High Opioid)	Communities That HEAL (CTH) intervention	Community Collations	N/R	None reported; study in progress
**Leonard, C, et al. (2019)** [48] (Leonard, C., et al. (2017) [49]; McCreight, M. S., et al. (2019) [38])	USA (Y- Veterans, Rural)	Transitions nurse program (TNP) is a Veteran-centered intervention carried out with a Transition Nurse (TN) in collaboration with a hospitalist site champion designed to improve transitional care for rural Veterans.	Pre-implementation Assessment Internal and External Facilitation Audit and Feedback	Using PRISM to evaluate site context yielded important insights of potential barriers and facilitators to implementation of a care coordination program and helped identify crucially important adaptations.	None reported; study in progress
**Liles, EG, et al. (2015)** [26] (Feldstein, A. C. & Glasgow, R. E., (2008) [4])	USA (N)	Centralized outreach program for colorectal cancer (CRC) screening	Direct Mailing Outreach Health-System Quality Improvement	Success was due in large part to the activation of three different domains within the PRISM framework: changing the delivery system design through centralizing screening efforts (implementation infrastructure); switching to a more accurate and feasible fecal test (external environment); and providing educational and electronic support (recipients of intervention). The combination of these actions resulted in a successful and sustained improvement in CRC screening rates.	Addressing barriers at multiple levels of a health system by changing the delivery system design to add a centralized outreach program, switching to a more accurate and easier to use fecal test, and providing educational and electronic support saw CRC screening rates improve 10% during program implementation and they continue to rise.
**Linke, SE, et al. (2020) **[50]	USA (N)	Quality improvement (QI) project that integrates Exercise is Medicine (EIM) into routine clinical practice	EHR Integration Audit and Feedback Plan-Do-Study-Act (PDSA) Cycles	N/R	None reported; study in progress
**Paniagua-Avila, A., et al. (2020) **[51]	Guatemala (Y- LMI Country)	A protocol-based hypertension treatment using a standardized algorithm; team-based collaborative care; health provider education; health coaching sessions; home blood pressure monitoring; blood pressure audit; and feedback	Clinician Trainings on Guidelines Interactive 2-day Workshop for Clinicians	N/R	None reported; study in progress
**Pittman, J. O. E., et al. (2020) **[52]	USA (Y- Medically uninsured)	Cognitive Rehabilitation and Exposure/Sorting Therapy (CREST), a compensatory cognitive training (CCT) modules designed to target cognitive impairments common in people with Hoarding Disorder (HD).	Training Individual and Group Supervision	N/R	There were significant changes in hoarding severity and clutter volume. Based on the initial 2 years of the program, funding was provided for expansion to cover additional San Diego County regions and hire more staff clinicians in year three. Preliminary data suggest that the CREST intervention can be successfully implemented in a community setting with positive results for older adults with HD.
**Satre, DD, et al. (2019) **[53]	USA (N)	A behavioral health specialist-delivered intervention in primary care with computerized screening and treatment for substance use disorder and depression and anxiety among people with HIV.	Clinician collaboration Training	N/R	None reported; study in progress
**Schneider, JL, et al. (2016) **[54]	USA (N)	Lynch syndrome screening for all newly diagnosed colon cancer patients	N/R	N/R	They completed 14 interviews with leaders/managers and staff representing involved clinical and health-plan departments. Although stakeholders supported the concept of universal screening, they identified several internal organizational) and external (environment) factors that promote or hinder implementation. Facilitating factors included perceived benefits of screening for patients and organization, collaboration between departments, and availability of organizational resources. Barriers were also identified, including: lack of awareness of guidelines, lack of guideline clarity, staffing and program “ownership” concerns, and cost uncertainties. Analysis also revealed nine important infrastructure-type considerations for successful implementation. Requirements for successful implementation may include interdepartmental collaboration and communication, patient and provider/ staff education, and significant infrastructure and resource support related to laboratory processing and systems for electronic ordering and tracking.
**Scholin, L., et al. (2019) **[55]	UK, Scotland (N)	Screening and brief intervention (SBI) consists of a short conversation focused on identifying problem drinking, motivating and facilitating reduction in drinking or abstinence to reduce the risk of harm.	N/R (N/R)	N/R	In several health boards, where reported maternal alcohol use was lower than expected, implementation leaders sought to optimize enquires about women’s alcohol use to facilitate honest disclosure. Strategies focused on having positive conversations, exploring pre-pregnancy drinking habits, and building a trusting relationship between pregnant women and midwives. Women's responses were encouraging and disclosure rates appeared improved, though with some unexpected variation over time. Adapting the intervention to the local context was also considered important. This is the first study to explore implementation leaders’ experiences of antenatal SBI delivery and identify possible changes in disclosure rates arising from the approach taken. Systems-informed evaluations of interventions in this setting that include consideration of unintended consequences are vital. A flexible, conversational approach to discussing alcohol with pregnant women was considered superior to formal tools for identifying who might benefit from interventions.
**Shields, N., et al. (2020) **[56]	Australia (Y- Disability)	FitSkills is a community based physical activity intervention to improve exercise participation among young people with disabilities.	Training and Orientation Log	N/R	None reported; study in progress
**Ssewamala, FM, et al. (2018) **[57] (McKay, M. M., et al. (2020) [58])	Uganda (Y- LMI Country)	The Multiple Family Group. a multi-generational intervention for youth at high contextual risk for behavioral challenges guided by the 4Rs and 2Ss	Parent Peers Community Health Workers Trainings and Workshops. Use of Champions Facilitation Educational Materials	N/R	Study in progress. Collaboration with global communities and governments plays a critical role in the adaptation, uptake, scalability, and sustainability of EBPs, and that the process of engagement and collaboration can be guided by conceptual frameworks [58].
**Stephens, TN, et al. (2014) **[59]	USA (Y- Underserved)	4Rs and 2Ss, a clinical intervention for groups of families with children (aged 7-11 years old) who meet the diagnostic criteria for oppositional defiant disorder or conduct disorder.	Learning Collaborative (LC) Training	The PRISM model was a useful framework to organize the data from the LC, which highlighted the aspects of program, external environment, implementation, sustainability infrastructure, and recipients that either promoted or hindered adoption of the 4Rs program beyond the initial period of active support afforded by the LC environment.	Clinics that were more proactive evidenced staff with advanced organizational skills were able to take advantage of the trainings and supports offered by the LC and fared better in their ability to adopt the intervention. The ability to adapt the intervention to the specific constraints of the clinics was a strong influence on continued use following the end of the LC. The dedicated service provided by the staff of the learning collaborative appeared to positively impact the rollout of the 4Rs Program. Problem-solving was a key component of the LC process. This translated to practical questions being answered in the sessions about what could be reasonably amended while maintaining fidelity. While success varied across clinics, there were a number of characteristics that were identified that are linked to successful adoption. These included factors related to their use of the program, their response to external environmental pressures, their mobilization of implementation and sustainability infrastructure, and recipient characteristics.
**Sullivan, J. L., et al. (2018)** [60]	USA (Y- Veterans)	Project Re-Engineered Discharge (RED) is a patient-centered, standardized intervention to improve hospital discharge processes	Multistep Toolkit-Guided Implementation	PRISM factors helped identify positive and negative influences/contextual factors on sites’ implementation of RED	Progress and adherence to the RED toolkit implementation steps and intervention components varied across study sites. Both higher- and lower-adherence sites were ultimately able to tailor and implement RED, in large part, because of its adaptability and flexibility. A majority of contextual factors identified were positive influences on sites’ implementation, including readiness to change (for example, to reduce high readmission rates), the presence of coordination across departments and specialties, and patient centeredness. However, the burden of undertaking a large intervention can also negatively influence and hinder program implementation.
**Woodbridge, M. W., et al. (2014) **[61]	USA (Y- Underserved)	First Step to Success is an early intervention program designed to help children in primary grades who are at risk for developing aggressive or antisocial behavior patterns.	N/R	N/R	A higher dosage of intervention days delivered successfully in the classroom was associated with higher academic engagement. Higher dosage of Home Base sessions was associated with higher academic engaged time. First Step was associated with improvements in students’ pro-social skills and reductions in problem behaviors. Teachers’ higher implementation fidelity was associated with greater student gains, and teachers’ delivery of higher dosage of First Step was associated with students’ greater academic engagement. From the qualitative data the participants did not think the intervention was overly complex or burdensome to implement. Educators advocated for a supportive infrastructure including high-quality, easily accessible training and regular technical assistance in the classroom. Participants recommended that teachers and students have access to the consistent support of a mentor or coach, who can encourage engagement in the EBP, problem solve about daily behavioral issues in the classroom, and provide immediate feedback about participant successes and challenges.
**Yakovchenko, V, et al. (2019) **[62]	USA (Y- Veterans)	Automated text messaging system (aTS) for patient self-management and allows clinical teams to monitor patient progress between in-person visits for hepatitis C virus (HCV) treatment.	Implementation Toolkit Support for local champion development Proactive outreach by the primary external facilitator	Considering the behavioral, social, organizational, and technical scale-up challenges that we documented, successful and sustained implementation of the aTS may require implementation strategies that operate at the clinic, provider, and patient levels.	Providers found the aTS appropriate with high potential for scale-up but not without difficulties in startup, patient selection and recruitment, and clinic workflow integration. Patients largely found the aTS easy to use and helpful; however, low perceived need for self-management support contributed to high declination. The aTS is a promising intervention for improving patient self-management; however, augmented approaches to implementation may be needed to support clinician buy-in and patient engagement. Despite positive perceptions of the aTS, patient enrollment was challenging; however, augmented facilitation resulted in the greater sustained engagement of patients once they enrolled. Among patients who used the aTS (texters) there was an indication of improved illness perception, health engagement, and patient activation.
**Zhang, R, et al. (2020) **[63] (Li, L., et al. (2020) [64])	China (Y- Rural)	Health education (HE) delivered through the community health service (Zhang et al. 2020) and Healthcare management for the aged (HMA) in basic public health service (BPHS) delivered by lay healthcare workers (LHWs) in primary health care (PHC) sectors (Li et al. 2020)	N/R	N/R	Less than 50% of residents who knew or utilized some item of HE. Distance to PHC sectors was associated with the knowledge of HE, gender and health insurance were associated with utilization of HE. Age, marital status, occupation region, and self-reported health were associated with satisfaction regarding HE. Barriers to HE delivery included defects in HE design, weak capacity in PHC sectors to provide HE, residents’ poor cooperation, lack of multi-sector cooperation, poor equipment and weak health system. Southwest China delivered HE in all PHC sectors. However, our study underlined many barriers to equalization of HE. To address those barriers and achieve HE quality improvement, comprehensive measures to improve capacity of PHC sectors, enhance multi-sector cooperation and strengthen health information systems are all urgent needs [63]. More than 85% of aged individuals had knowledge and utilization of HMA, and over 94% of these respondents were satisfied with HMA. Challenges in HMA delivery included weakness (unmet items and lack of appropriate assessment indicators) in HMA design, low capacity of PHC sectors and competency of LHWs to deliver HMA, poor health literacy of aged individuals, insufficient funds, and a lack of multi-sector cooperation. Though significant achievements in HMA were observed, this study highlighted the challenges in further quality improvement of HMA delivery program in Southwest China. The “older-person-centered and integrated care” model provided a good theory to improve the quality of HMA by reinforcing the needs-based HMA design, building a comprehensive assessment strategy, improving the capacity of PHC sectors and the LHWs’ competency, and strengthening multi-sector cooperation [64].

*Y* yes, *N* no, *LMI* low- and middle-income country, *N/R* not reported, *PRISM* Practical, Robust Implementation and Sustainability Model, *EHR* electronic health record, *EBP* evidence-based practice

The target populations for the studies were mainly focused on the providers or delivery agents (*n*= 20), but most also included the setting level (*n*=16) and patient or community (*n*=15 for both groups). One study targeted payors and another targeted policy makers (Table 3). Most studies that integrated PRISM were conducted in the clinical out-patient setting (*n*=13) with a minority of studies being conducted in community settings (*n*=4). Additional settings identified were clinical in-patient (*n*=3), schools (*n*=2), or as part of a national health initiative (*n*=2). Two studies were conducted in other settings (i.e., a care transition context and a regional evaluation). The study design varied greatly across studies with seven being stand-alone protocol papers, five being randomized controlled trials, four quasi-experimental pre/post designs, three case studies, one cohort study, and one narrative piece (Table 3). Of note, nine were classified as having a design different from above such as qualitative implementation study, cross-sectional study, and adaptation study. The type of study varied from nine that were specifically implementation focused; six that were type 2 hybrid effectiveness-implementation; three efficacy studies; and two were effectiveness studies. Five studies were classified as pilot studies and five did not fit into any of the study type categories. The most frequently used methods used were qualitative (*n*=8), mixed methods (*n*=7) and multi-method (*n*=6). Only two studies reported strictly quantitative methods. In terms of socioecologic level, 14 studies used setting, four individual, three delivery agent, and two had multiple levels at which randomization and/or data collection happened.

**Table 3:** Summary of domain use of Practical, Robust Implementation and Sustainability Model (PRISM) and Reach, Effectiveness, Adoption, Implementation, and Maintenance (RE-AIM) frameworks (*N*=23)

Citation	Target population	Study setting	Study design	Methods used	Level of analysis	Program/intervention (O)	Program/intervention (P)	Recipients (O)	Recipients (P)	Implementation and sustainability infrastructure	External environment	RE-AIM used
Ameling, JM, et al. (2014) [35]	I, CM, DA, S	COP	O	QUAL	I	X	X	X	X	X	X	
Ayele, RA, et al. (2017) [36]	I, CM, DA	CIP, COP	CS	QUAL	S			X	X	X	X	
Ekawati, FM, et al. (2019) [39]	I, CM, DA, S	COP	SAP	MIXED	S	X			X			
Esses, SA, et al. (2019) [41]	I, CM, DA	CIP	RCT, PP	QUAN	I	X	X	X	X			
Gopalan, G, et al. (2016) [42]	I, CM, DA	COM	O	MIXED	ML	X	X	X	X	X	X	IM
Gopalan, G, et al. (2014) [45]	DA, S	COP	O	QUAL	S	X		X	X	X	X	R, AS, AST, IM
Henderson, V, et al. (2020) [46]	I, CM, S	COP	PP	QUAN	I	X	X		X	X		
Knudsen, HK, et al. (2020) [47]	DA	COM	NP, SAP	MULTI	DA	X		X		X	X	
Leonard, C, et al. (2019) [48]	I, CM, DA, S	CT	CS	MIXED	S	X	X	X	X	X	X	
Liles, EG, et al. (2015) [26]	DA, S	COP	O	QUAL	S	X		X		X	X	E, IM
Linke, SE, et al. (2020) [50]	I, CM, DA, S	COP	SAP	MULTI	S	X	X	X		X	X	
Paniagua-Avila, A, et al. (2020) [51]	I, CM, DA, S	COP	RCT, SAP	MULTI	S	X	X			X		
Pittman, JOE, et al. (2020) [52]	I, CM	COM	PP	MIXED	I	X	X	X	X	X	X	
Satre, DD, et al. (2019) [53]	I, CM, DA, S	COP	PP, SAP	MIXED	S	X	X		X			
Schneider, JL, et al. (2016) [54]	DA	COP	O	QUAL	DA	X		X		X	X	
Scholin, L, et al. (2019) [55]	DA, S	NHI, COP	O	QUAL	DA	X		X		X	X	
Shields, N, et al. (2020) [56]	I, CM, S	COM	RCT, COH, SAP	MULTI	S	X	X	X	X	X	X	
Ssewamala, FM, et al. (2018) [57]	I, CM, DA, S	SCH	RCT, SAP	MIXED	S	X			X	X		AS
Stephens, TN, et al. (2014) [59]	DA, S	COP	O	QUAL	S	X	X	X		X	X	R, AS, AST, IM, MI, MS
Sullivan, JL, et al. (2018) [60]	DA, S	CIP	O	QUAL	S	X	X	X	X	X		R, IM
Woodbridge, MW, et al. (2014) [61]	DA, S	SCH	CS	MULTI	S	X	X		X	X	X	
Yakovchenko, V, et al. (2019) [62]	I, CM, DA, S	COP	RCT	MIXED	S	X		X	X	X		
Zhang, R, et al. (2020) [63]	I, CM, DA	NHI, RE	O	MULTI	ML	X	X	X	X	X	X	

Target population: *O* organization, *I* individual (patient/participant), *CM* community member, *DA* delivery agent/provider, *S* setting

Study setting: *COP* clinical out-patient, *CIP* clinical in-patient, *COM* community, *SCH* school, *NHI* national health initiative, *CT* care transitions, *RE* regional evaluation

Study design: *NP* narrative piece, *CS* case study, *COH* cohort study, *PP* pre/post, *RCT* randomized controlled trial, *SAP* stand-alone protocol paper, *O* other

Methods used: *QUAL* qualitative, *MIXED* mixed methods, *QUAN* quantitative, *MULTI* multiple methods

Level of randomization/data collection: *I* individual (patient/participant), *ML* multi-level, *DA* delivery agent (provider/implementation staff), *S* setting

RE-AIM used: *R* reach, *E* effectiveness, *AS* adoption: setting, *AST* adoption: staff, *IM* implementation, *MI* maintenance: individual, *MS* maintenance: setting

### PRISM use

PRISM was used primarily for study evaluation (*n*=19), but also for planning and development (*n*=10), and implementation (*n*=10). Obviously, many addressed multiple implementation stages. Four studies addressed sustainment, and only one study addressed dissemination (Additional file 3). The research team identified two studies, Ayele, R. A., et al. [36] and Leonard et al. [48], that used PRISM for planning and development, implementation, and evaluation. Woodbridge, M. W., et al. [61] was found to be an exemplar in evaluation and sustainment. There were no exemplar studies identified for dissemination.

Table 3 provides further details on the extent to which studies included the various context domains of PRISM and Table 4 shows what sub-domains were addressed. Twenty-two studies included perspectives on the Program/Intervention from the organizational lens, with nearly three-quarters addressing barriers for frontline staff. There were 15 studies including Program/Intervention from the patient perspective focusing on patient barriers, being patient-centered, and addressing service and access. Recipient characteristics at the organizational level (*n*=18) focused on management support and communication as well as shared goals and cooperation. Recipient patient characteristics reported (*n*=16) were primarily related to demographics. Most studies (*n*=21) included Implementation and Sustainability Infrastructure and performance data was the most frequent sub-domain reported within this factor. Studies addressing the external environment domain (*n*=19) were primarily focused on community resources.

**Table 4:** Inclusion and operationalization of Practical, Robust Implementation and Sustainability Model (PRISM) elements across all articles included in review by dimension and evaluation criteria: 2008–2020

PRISM elements	Average inclusion, ***n***	Operationalization of elements, ***n***
**Perspectives on program/intervention**		
*Organizational perspective (n=22)*		
• Readiness	12	Qualitative: 11 Quantitative 3 Mixed methods: 4 Multi-methods: 3 Narrative: 1
• Strength of the evidence base	8	
• Addresses barriers of frontline staff	16	
• Coordination across departments and specialties	9	
• Burden (complexity and cost)	11	
• Usability and adaptability	10	
• Trialability and reversibility	0	
• Ability to observe results	9	
*Patient perspective (n=15)*		
• Patient centeredness	6	Qualitative: 6 Quantitative 2 Mixed methods: 2 Multi-methods: 4 Narrative: 1
• Provides patient choices	2	
• Addresses patient barriers	6	
• Seamlessness of transition between program elements	2	
• Service and access	6	
• Burden (complexity and cost)	5	
• Feedback of results	4	
**Recipients**		
*Organizational characteristics (n=18)*		
• Organizational health and culture	8	Qualitative: 10 Quantitative 3 Mixed methods: 2 Multi-methods: 2 Narrative: 1
• Management support and communication	9	
• Shared goals and cooperation	9	
• Clinical leadership	4	
• Systems and training	8	
• Data and decision support	4	
• Staffing and incentives	6	
• Expectation of sustainability	5	
*Patient characteristics (n=16)*		
• Demographics	12	Qualitative: 5 Quantitative 5 Mixed methods: 3 Multi-methods: 2 Narrative: 1
• Disease burden	10	
• Competing demands	5	
• Knowledge and beliefs	5	
**Implementation and sustainability infrastructure** (*n*=21)		
• Performance data	13	Qualitative: 11 Quantitative 2 Mixed methods: 4 Multi-methods: 3 Narrative: 1
• Dedicated team	11	
• Adopter training and support	12	
• Relationship and communication with adopters (bridge researchers)	3	
• Adaptable protocols and procedures	11	
• Facilitation of sharing of best practices	6	
• Plan for sustainability	8	
**External environment** (*n*=19)		
• Payor satisfaction	4	Qualitative: 13 Quantitative 2 Mixed methods: 1 Multi-methods: 2 Narrative: 1
• Competition	3	
• Regulatory environment	8	
• Reimbursement	6	
• Community resources	9	

In terms of the operationalization of the PRISM context domains, most domains were operationalized using qualitative methods followed by quantitative, mixed methods, and muti-methods approaches and only one study used a narrative approach. The specific distribution of the methods for each domain is provided in Table 4. Specific techniques used for data collection about each domain were not as consistently reported but a general review of themes identified interviews, focus groups, and surveys as the most common approaches of data collection with a few studies reporting the use of more innovative approaches such as brainwriting or process mapping) (data not shown). When rating the degree of operationalization of the PRISM domains across the various stages of use of PRISM (i.e., planning/development, dissemination, implementation, evaluation, and sustainment) we found that average ratings were similar across the stages and ranged from 2.3 to 3.1 on the scale of 0 to 5 where 0 was poor and 5 excellent. Operationalization of the PRISM domains for supporting sustainment was rated on average the lowest and implementation the highest. Lower ratings were due to a lack of specificity on how the PRISM domains were operationalized for the given stage.

Only eight included studies provided specific lessons learned about the contribution of PRISM to planning, implementation, and/or evaluation (Table 2). The following key themes were identified when reviewing the lessons learned: (1) PRISM was useful in supporting pre-implementation planning and adaptation of interventions to multilevel contexts [42, 45, 48]; (2) PRISM was feasible and useful in supporting the implementation of interventions in multilevel contexts [26, 46]; and (3) PRISM with its multilevel consideration of determinants was helpful in organizing multilevel influencers of implementation [59, 60].

There were six studies that included all six contextual domains. Despite the RE-AIM outcomes being an explicit part of the original PRISM model (Fig. 1 and Table 3), only two studies directly connected individual PRISM domains with RE-AIM outcomes, and another four studies included RE-AIM outcomes without directly connecting them to the PRISM context domains. Even the two papers in which a connection between the context domain and RE-AIM was made, the studies did not attempt to explicitly test relationships between the contextual factors and RE-AIM outcomes. Of the six studies that included RE-AIM, all but one addressed implementation, three addressed reach, three adoption at the staff and/or setting level, one included effectiveness, and one maintenance at the individual and setting level. One included all RE-AIM dimensions except effectiveness and no studies included all five RE-AIM outcomes.

Few studies combined PRISM with another TMF (*n*=7) and even fewer described the adaptation of PRISM for the study context (*n*=2). TMFs that were used in combination with PRISM included the expanded framework for reporting adaptations and modifications to evidence-based interventions (FRAME) [65], the learning evaluation [66], the Social Ecological Model [67], and Lean Six Sigma [68]. Adaptations to PRISM focused on the modifications of domains to align with the context of the study. Goplan et al. adapted and tailored PRISM to reflect the context of their 4Rs and 2Ss for strengthening families intervention [45] and Knudsen et al. modified how the implementation and sustainability infrastructure and external environment are structured based on their community and collation partners [47].

## Discussion

PRISM was developed to specify multi-level contextual factors related to implementation research outcomes included in the Reach, Effectiveness, Adoption, Implementation, Maintenance (RE-AIM) framework [4, 27, 69]. Our review summarized published reports of use of PRISM from 2002 to 2020 and attempted to report on findings related to relationships between PRISM contextual factors and RE-AIM outcomes. Nearly 200 articles referenced PRISM, used it to inform or direct their research, or integrated it within the research design—with the numbers growing over the past decade. The studies reflected a diverse body of literature that applied PRISM across the stages of intervention planning, implementation, and evaluation. While 23 studies (represented by 32 articles) integrated PRISM within their research methodology, very few included PRISM contextual factors and RE-AIM outcomes together. None made an explicit attempt to test the connection between contextual domains and RE-AIM outcomes, making it difficult to determine the relationships between specific contextual constructs and unique implementation outcomes.

We noted that the purpose and use of PRISM varied across included studies and the level of specificity on how PRISM was integrated and measured was often lacking, making it difficult to compare findings across studies. Similarly, it was challenging to rate the degree and quality of use of PRISM as there were few studies using or reporting on the PRISM sub-domains comprehensively. More guidance on how to use PRISM especially as it relates to its sub-domains should be provided for future studies. Furthermore, measures should be linked to specific PRISM domains and sub-domains to support a more comprehensive use of the model.

Studies that have integrated PRISM in their research methodology reflect a body of literature that is relatively early in its development. As a result, few studies reported using PRISM as a framework to describe potential relationships between context and outcomes. Indeed, many of the studies have used PRISM in planning and characterizing dissemination and implementation settings and “recipients” (participants, implementers, and organizations). A large proportion of these studies examined implementation and sustainability infrastructure issues when planning for intervention implementation. Explicitly reporting on this dimension is seen as one of the strengths of PRISM (explicit focus on this construct is relatively unique among context-oriented implementation science frameworks) and conceptually is likely to be strongly related to sustainment, although this remains to be tested. We were surprised to find that 78% of the studies addressed health equity in some way. This focus was primarily operationalized by the primary focus of the studies on underserved populations. We believe that the main reason for this high percentage of health equity focus is due to the focus of the RE-AIM outcomes on representativeness which lends itself to health equity applications [20]. This trend should be further broadened to include the standard use of equity-related factors in each of the PRISM domains.

The application, operationalization, and assessment of every construct within PRISM including the implementation outcomes reflected in RE-AIM within a single study can be challenging [70]. Only a small percentage of originally identified articles met the criteria for the “integrated” use of PRISM. Given several new developments related to implications and use of RE-AIM to address equity issues, which is a key part of the PRISM framework, there is likely to be a lot more in the near future [27, 71–73]. Thus, it is timely to review what has been learned and to make recommendations for future applications of PRISM.

Key findings from our review are that (1) PRISM has been primarily used in outpatient clinical settings and in the US; (2) it has been used to study a variety of issues and conditions using a wide range of experimental designs and often using mixed methods; (3) most studies have reported on half or more of the PRISM domains and over half of the studies reported on at least 5 of the 6 domains; (4) PRISM contextual components most frequently assessed were the Program/Intervention characteristics from the organizational perspective, the Implementation and Sustainability Infrastructure, and the Organizational and Individual Recipient characteristics; (5) PRISM contextual components were most frequently operationalized using qualitative methods followed by quantitative, mixed-, and multi-method approaches; and (6) for the RE-AIM aspects of PRISM, the outcomes most often reported were Implementation and Maintenance.

Compared to the widespread use of RE-AIM, there has been a modest uptake of PRISM, especially until the last few years. There are multiple potential reasons for this finding including that the original article was not in a high-impact journal and was published before Open Access and citation services dramatically increased article accessibility. Other reasons likely include that the National RE-AIM Working Group until very recently has not promoted PRISM and that it has not been taught as part of the major training programs in D&I science. Finally, to date, most studies have treated PRISM and RE-AIM as separate frameworks rather than reporting them as directly related, and PRISM as being an expansion of RE-AIM [27, 71, 74]. These findings present both a need and an opportunity for future researchers to better incorporate the full PRISM framework into their studies. In this review, only six of the 23 studies included any RE-AIM outcomes, despite these outcomes being an explicit part of the original model (Table 3). More consistent inclusion of RE-AIM outcomes would also allow for more robust assessments of how PRISM domains impact implementation outcomes, including formal mediation or moderation analysis.

We found that due to the lack of consistency and specificity in the use of PRISM and reporting on the operationalization of the PRISM domains, it was challenging to synthesize information across studies on the contributions of PRISM to support planning, implementation, and evaluation. When reviewing the eight studies that identified specific lessons learned about the use of PRISM, we noted that these studies found PRISM useful in supporting planning, implementation, and evaluation efforts especially due its multilevel, contextual orientation. More comprehensive and proactive use of PRISM in future studies will allow the broader analysis and synthesis of conclusions and lessons learned.

## Strengths and limitations

This review has both strengths and limitations. Limitations include that the review was limited to English language reports and to published research. It is possible that creative work and important examples of cultural adaptations using PRISM may have been missed with these decisions. The resulting 23 studies, while a large enough sample to draw conclusions and suggest directions for future application, is too small to conduct meta-analyses or draw definitive conclusions. Furthermore, our review relied on the initial classification of papers regarding the use of PRISM based on the information provided in the published papers that were identified through our multi-step search and validation process. It is possible that contacting the corresponding authors of these papers could have yielded more information about the use and resulted in different classification of these papers (e.g., ‘integrated’ instead of ‘directed’ use). This paper was also solely focused on describing pattern of use for studies that were classified as integrated use. Future papers could explore how PRISM and other TMFs have been used I n Framing and Directed studies. Finally, studies provided less consistent information on the operationalization of each PRISM domain which limited our ability to systematically report and synthesize information about this aspect. In this paper, we were able to include information about the methods used to operationalize domains and the most frequent techniques used for data collection.

Strengths of this review include following PRISMA recommendations and reporting standards; the use of multiple reviewers and coders at all review stages; and the use of the application categories previously developed by Field et al. [29] to justify and explain exclusions. We also used multiple search strategies, including reverse citation approaches and recommendations from prominent investigators- these proved effective in increasing the number of relevant articles.

### Next steps and recommendations

We summarize key recommendations for research and practice directions:

*For research*, we recommend:

1. The development and validation of more quantitative measures of PRISM, especially those that meet pragmatic [75] and the PAPERS [76] criteria. This will allow greater mixed-methods research on PRISM and understanding of various linkages.
2. The use of common and where applicable, standardized PRISM definitions, assessments, and criteria. We note examples of mixed methods research with PRISM, including survey and qualitative interview guides as well as a new interactive PRSIM assessment and feedback tool that will soon be available on the re-aim.org website.
3. While preferred to advance implementation science, for pragmatic use it is not necessary to use all PRISM components or to use PRISM at all program time points (i.e., pre-implementation, implementation, sustainment [70]). When not feasible, authors should briefly and transparently state why certain components were not used or why PRISM was only used at one time point.
4. More investigations and transparent reporting are needed that (a) compare PRISM with other TMFs and create clear cross-walks between PRISM and other TMFs; (b) combine PRSIM with other models; and (c) adapt PRISM to diverse contexts and content areas.
5. Patient and community member issues involving (a) characteristics components of PRISM can be highlighted more; including especially social determinants of health and other equity-related issues; and (b) perceptions of the beneficiary of the intervention (e.g., patient-centeredness, trialability, relative advantage/etc.)
6. Consider using PRISM in more diverse settings including community, school, worksite, and other non-clinical contexts, especially including low resource settings and low- and middle-income countries.

*For implementation practice*, we recommend:

1. Reports on how PRISM is used with different types of implementation partners, in multi-sector research, and for team science, including the time involved and lessons learned.
2. Use of PRISM in logic models and to develop participatory implementation strategies that can help to address priority outcomes.
3. As discussed in the section on clarification, reviewing PRISM terminology to make it more user-friendly and relevant to the context of the implementation practice—for example changing some terms such as “patients” or “recipients.”
4. Development and usability evaluations of interactive tools and resources including videos that illustrate and guide PRISM use.

## Conclusions

Although initial uptake of PRISM was slow after its initial publication in 2008, usage has increased and over 50 studies were published in 2019 and the most of 2020. While there are opportunities to further expand integrated use of PRISM and more explicit reports on linkage to RE-AIM outcomes, this review has produced learnings and recommendations for future directions. It is hoped this publication will encourage more sophisticated use and reporting on PRISM to inform both research and practice on contextual factors in implementation.

## Supplementary Information

**Additional file 1.** Abstraction form.

**Additional file 2.** All papers included in the Practical, Robust Implementation and Sustainability Model review with level of use and search source.

**Additional file 3.**

## Data Availability

The datasets analyzed during the current study available from the corresponding author on reasonable request. A separate protocol was to prepared for this study.

## References

[1] Glasgow RE, Chambers D (2012). Developing robust, sustainable, implementation systems using rigorous, rapid and relevant science. Clin Transl Sci.

[2] Brownson RC, Colditz GA, Proctor EK (2018). Dissemination and implementation research in health: translating science to practice.

[3] May CR, Johnson M, Finch T (2016). Implementation, context and complexity. Implement Sci.

[4] Feldstein AC, Glasgow RE (2008). A practical, robust implementation and sustainability model (PRISM) for integrating research findings into practice. Jt Comm J Qual Patient Saf.

[5] Tabak RG, Khoong EC, Chambers DA, Brownson RC (2012). Bridging research and practice: models for dissemination and implementation research. Am J Prev Med.

[6] Birken SA, Rohweder CL, Powell BJ, Shea CM, Scott J, Leeman J (2018). T-CaST: an implementation theory comparison and selection tool. Implement Sci.

[7] Brownson RC, Kumanyika SK, Kreuter MW, Haire-Joshu D (2021). Implementation science should give higher priority to health equity. Implement Sci.

[8] Kislov R, Pope C, Martin GP, Wilson PM (2019). Harnessing the power of theorising in implementation science. Implement Sci.

[9] Wensing M, Sales A, Wilson P, Armstrong R, Kislov R, Rankin NM (2021). Implementation Science and Implementation Science Communications: a refreshed description of the journals’ scope and expectations. Implement Sci.

[10] Nilsen P, Bernhardsson S (2019). Context matters in implementation science: a scoping review of determinant frameworks that describe contextual determinants for implementation outcomes. BMC Health Serv Res.

[11] Movsisyan A, Arnold L, Evans R, Hallingberg B, Moore G, O’Cathain A (2019). Adapting evidence-informed complex population health interventions for new contexts: a systematic review of guidance. Implement Sci.

[12] Pfadenhauer LM, Gerhardus A, Mozygemba K, Lysdahl KB, Booth A, Hofmann B (2017). Making sense of complexity in context and implementation: the Context and Implementation of Complex Interventions (CICI) framework. Implement Sci.

[13] Damschroder LJ (2020). Clarity out of chaos: use of theory in implementation research. Psychiatry Res.

[14] Moullin JC, Dickson KS, Stadnick NA, Rabin B, Aarons GA (2019). Systematic review of the Exploration, Preparation, Implementation, Sustainment (EPIS) framework. Implement Sci.

[15] Squires JE, Graham ID, Hutchinson AM, Michie S, Francis JJ, Sales A (2015). Identifying the domains of context important to implementation science: a study protocol. Implement Sci.

[16] Schroeder D, Luig T, Finch TL, Beesoon S, Campbell-Scherer DL (2022). Understanding implementation context and social processes through integrating Normalization Process Theory (NPT) and the Consolidated Framework for Implementation Research (CFIR). Implement Sci Commun.

[17] Watson DP, Adams EL, Shue S, Coates H, McGuire A, Chesher J (2018). Defining the external implementation context: an integrative systematic literature review. BMC Health Serv Res.

[18] Moore GF, Evans RE, Hawkins J, Littlecott H, Melendez-Torres GJ, Bonell C (2019). From complex social interventions to interventions in complex social systems: future directions and unresolved questions for intervention development and evaluation. Evaluation (Lond).

[19] Chambers DA, Glasgow RE, Stange KC (2013). The dynamic sustainability framework: addressing the paradox of sustainment amid ongoing change. Implement Sci.

[20] Shelton RC, Chambers DA, Glasgow RE (2020). An extension of RE-AIM to enhance sustainability: addressing dynamic context and promoting health equity over time. Front Public Health.

[21] Aarons GA, Green AE, Palinkas LA, Self-Brown S, Whitaker DJ, Lutzker JR (2012). Dynamic adaptation process to implement an evidence-based child maltreatment intervention. Implement Sci.

[22] Proctor E, Silmere H, Raghavan R, Hovmand P, Aarons G, Bunger A (2011). Outcomes for implementation research: conceptual distinctions, measurement challenges, and research agenda. Adm Policy Ment Health Ment Health Serv Res.

[23] Rabin BA, Brownson RC, Haire-Joshu D, Kreuter MW, Weaver NL (2008). A glossary for dissemination and implementation research in health. J Public Health Manag Pract.

[24] Beck A, Bergman DA, Rahm AK, Dearing JW, Glasgow RE (2009). Using implementation and dissemination concepts to spread 21st-century well-child care at a health maintenance organization. Perm J.

[25] Nilsen P (2015). Making sense of implementation theories, models and frameworks. Implement Sci.

[26] Liles EG, Schneider JL, Feldstein AC, Mosen DM, Perrin N, Rosales AG (2015). Implementation challenges and successes of a population-based colorectal cancer screening program: a qualitative study of stakeholder perspectives. Implement Sci.

[27] Glasgow RE, Harden SM, Gaglio B, Rabin B, Smith ML, Porter GC (2019). RE-AIM planning and evaluation framework: adapting to new science and practice with a 20-year review. Front Public Health.

[28] Bergström A, Ehrenberg A, Eldh AC, Graham ID, Gustafsson K, Harvey G (2020). The use of the PARIHS framework in implementation research and practice-a citation analysis of the literature. Implement Sci.

[29] Field B, Booth A, Ilott I, Gerrish K (2014). Using the Knowledge to Action Framework in practice: a citation analysis and systematic review. Implement Sci.

[30] Harden SM, Gaglio B, Shoup JA, Kinney KA, Johnson SB, Brito F (2015). Fidelity to and comparative results across behavioral interventions evaluated through the RE-AIM framework: a systematic review. Syst Rev.

[31] Gaglio B, Shoup JA, Glasgow RE (2013). The RE-AIM framework: a systematic review of use over time. Am J Public Health.

[32] Harris P, Taylor R, Thielke R, Payne J, Gonzalez N, Conde J (2009). A metadata-driven methodology and workflow process for providing translational research informatics support. J Biomed Inform.

[33] Harris PA, Taylor R, Minor BL, Elliott V, Fernandez M, O’Neal L (2019). The REDCap consortium: building an international community of software platform partners. J Biomed Inform.

[34] Braveman PAE, Orleans T, Proctor D, Plough A (2017). What is health equity?.

[35] Ameling JM, Ephraim PL, Bone LR, Levine DM, Roter DL, Wolff JL (2014). Adapting hypertension selfmanagement interventions to enhance their sustained effectiveness among urban African Americans. Fam Commun Health..

[36] Ayele RA, Lawrence E, McCreight M, Fehling K, Peterson J, Glasgow RE (2017). Study protocol: improving the transition of care from a non-network hospital back to the patient's medical home. BMC Health Serv Res..

[37] Ayele RA, Lawrence E, McCreight M, Fehling K, Glasgow RE, Rabin BA, et al. Perspectives of Clinicians, Staff, and Veterans in Transitioning Veterans from non-VA Hospitals to Primary Care in a Single VA Healthcare System. J Hosp Med. 2019;14(3):E1–e7. 10.12788/jhm.3320 PMC7064299 31634102

[38] McCreight MS, Rabin BA, Glasgow RE, Ayele RA, Leonard CA, Gilmartin HM (2019). Using the Practical, Robust Implementation and Sustainability Model (PRISM) to qualitatively assess multilevel contextual factors to help plan, implement, evaluate, and disseminate health services programs. Transl Behav Med..

[39] Ekawati FM, Licqurish S, Emilia O, Gunn J, Brennecke S, Lau P (2019). Developing management pathways for hypertensive disorders of pregnancy (HDP) in Indonesian primary care: a study protocol. Reprod Health..

[40] Ekawati FM, Emilia O, Gunn J, Licqurish S, Lau P (2020). The elephant in the room: an exploratory study of hypertensive disorders of pregnancy (HDP) management in Indonesian primary care settings. BMC Fam Pract..

[41] Esses SA, Small S, Rodemann A, Hartman ME (2019). Post-Intensive Care Syndrome: Educational Interventions for Parents of Hospitalized Children. Am J Crit Care..

[42] Gopalan G (2016). Feasibility of improving child behavioral health using task-shifting to implement the 4Rs and 2Ss program for strengthening families in child welfare. Pilot Feasibility Stud..

[43] Gopalan G, Hooley C, Winters A, Stephens T (2019). Perceptions Among Child Welfare Staff when Modifying A Child Mental Health Intervention to be Implemented in Child Welfare Services. Am J Community Psychol..

[44] Hooley C, Winters AM, Pisciotta C, Gopalan G. Caregiver-relevant perspectives from a multi-stakeholder collaborative advisory board on adapting a child mental health intervention to be delivered in child-welfare settings. J Public Child Welf. 2020:1–23. 10.1080/15548732.2020.1724238 PMC8261574 34248440

[45] Gopalan G, Franco LM, Dean-Assael K, McGuire-Schwartz M, Chacko A, McKay M (2014). Statewide implementation of the 4 Rs and 2 Ss for strengthening families. J Evid Based Soc Work.

[46] Henderson V, Tossas-Milligan K, Martinez E, Williams B, Torres P, Mannan N (2020). Implementation of an integrated framework for a breast cancer screening and navigation program for women from underresourced communities. Cancer.

[47] Knudsen HK, Drainoni M-L, Gilbert L, Huerta TR, Oser CB, Aldrich AM (2020). Model and approach for assessing implementation context and fidelity in the HEALing Communities Study. Drug Alcohol Depend.

[48] Leonard C, Gilmartin H, McCreight M, Kelley L, Lippmann B, Mayberry A (2019). Operationalizing an implementation framework to disseminate a care coordination program for rural veterans. J Gen Intern Med.

[49] Leonard C, Lawrence E, McCreight M, Lippmann B, Kelley L, Mayberry A, et al. Implementation and dissemination of a transition of care program for rural veterans: a controlled before and after study. Implement Sci. 2017;12(1):123. 10.1186/s13012-017-0653-1 PMC5651587 29058640

[50] Linke SE, Kallenberg GR, Kronick R, Tai-Seale M, De-Guzman K, Rabin B. Integrating “Exercise Is Medicine” into primary care workflow: a study protocol. Transl Behav Med. 2020. 10.1093/tbm/ibaa088 PMC8599878 32945881

[51] Paniagua-Avila A, Fort MP, Glasgow RE, Gulayin P, Hernández-Galdamez D, Mansilla K (2020). Evaluating a multicomponent program to improve hypertension control in Guatemala: study protocol for an effectiveness-implementation cluster randomized trial. Trials..

[52] Pittman JOE, Davidson EJ, Dozier ME, Blanco BH, Baer KA, Twamley EW, et al. Implementation and evaluation of a community-based treatment for late-life hoarding. Int Psychogeriatr. 2020:1–10. 10.1017/S1041610220000241 PMC7483985 32131916

[53] Satre DD, Anderson AN, Leibowitz AS, Levine-Hall T, Slome S, Flamm J, et al. Implementing electronic substance use disorder and depression and anxiety screening and behavioral interventions in primary care clinics serving people with HIV: Protocol for the Promoting Access to Care Engagement (PACE) trial. Contemp Clin Trials. 2019;84. 10.1016/j.cct.2019.105833 PMC6760257 31446142

[54] Schneider JL, Davis J, Kauffman TL, Reiss JA, McGinley C, Arnold K (2016). Stakeholder perspectives on implementing a universal Lynch syndrome screening program: a qualitative study of early barriers and facilitators. Genet Med..

[55] Schölin L, Fitzgerald N (2019). The conversation matters: a qualitative study exploring the implementation of alcohol screening and brief interventions in antenatal care in Scotland. BMC Pregnancy Childbirth..

[56] Shields N, Willis C, Imms C, Prendergast LA, Watts JJ, van Dorsselaer B, et al. FitSkills: protocol for a stepped wedge cluster randomised trial of a community-based exercise programme to increase participation among young people with disability. BMJ Open. 2020;10(7):e037153. 10.1136/bmjopen-2020-037153 PMC7348474 32641337

[57] Ssewamala FM, Sensoy Bahar O, McKay MM, Hoagwood K, Huang KY, Pringle B (2018). Strengthening mental health and research training in Sub-Saharan Africa (SMART Africa): Uganda study protocol. Trials..

[58] McKay MM, Sensoy Bahar O, Ssewamala FM. Implementation science in global health settings: Collaborating with governmental & community partners in Uganda. Psychiatry Res. 2020;283. 10.1016/j.psychres.2019.112585 PMC6954316 31590906

[59] Stephens TN, McGuire-Schwartz M, Rotko L, Fuss A, McKay MM (2014). A learning collaborative supporting the implementation of an evidence-informed program, the “4Rs and 2Ss for children with conduct difficulties and their families”. J Evid Based Soc Work.

[60] Sullivan JL, Shin MH, Engle RL, Yaksic E, Lukas CV, Paasche-Orlow MK (2018). Evaluating the implementation of project Re-Engineered Discharge (RED) in five Veterans Health Administration (VHA) hospitals. Jt Comm J Qual Patient Saf.

[61] Woodbridge MW, Sumi WC, Yu J, Rouspil K, Javitz HS, Seeley JR (2014). Implementation and Sustainability of an Evidence-Based Program Lessons Learned From the PRISM Applied to First Step to Success. J Emot Behav Disord..

[62] Yakovchenko V, Hogan TP, Houston TK, Richardson L, Lipschitz J, Petrakis BA, et al. Automated text messaging with patients in Department of Veterans Affairs specialty clinics: Cluster randomized trial. J Med Internet Res. 2019;21(8). 10.2196/14750 PMC6729116 31444872

[63] Zhang R, Chen Y, Liu S, Liang S, Wang G, Li L, et al. Progress of equalizing basic public health services in Southwest China--- health education delivery in primary healthcare sectors. BMC Health Serv Res. 2020;20(1):247. 10.1186/s12913-020-05120-w PMC7092608 32209085

[64] Li L, Zhang R, Chen Y, Deng H, Liu S, Wang G, et al. Achievements and challenges in health management for aged individuals in primary health care sectors: a survey in Southwest China. BMC Public Health. 2020;20(1):338. 10.1186/s12889-020-8210-2 PMC7077106 32178646

[65] Wiltsey Stirman S, Baumann AA, Miller CJ (2019). The FRAME: an expanded framework for reporting adaptations and modifications to evidence-based interventions. Implement Sci.

[66] Balasubramanian BA, Cohen DJ, Davis MM, Gunn R, Dickinson LM, Miller WL (2015). Learning evaluation: blending quality improvement and implementation research methods to study healthcare innovations. Implement Sci.

[67] Sallis JF, Owen N, Fisher EB (2008). Ecological models of health behavior. Health behavior and health education: theory, research, and practice.

[68] Raval SJ, Kant R (2017). Study on Lean Six Sigma frameworks: a critical literature review. Int J Lean Six Sigma.

[69] Glasgow RE, Vogt TM, Boles SM (1999). Evaluating the public health impact of health promotion interventions: the RE-AIM framework. Am J Public Health.

[70] Glasgow RE, Estabrooks PE (2018). Pragmatic applications of RE-AIM for health care initiatives in community and clinical settings. Prev Chronic Dis.

[71] Holtrop JS, Estabrooks PA, Gaglio B, Harden SM, Kessler RS, King DK (2021). Understanding and applying the RE-AIM framework: Clarifications and resources. J Clin Transl Sci.

[72] Matlock DD, Fukunaga MI, Tan A, Knoepke C, McNeal DM, Mazor KM (2020). Enhancing success of medicare’s shared decision making mandates using implementation science: examples applying the Pragmatic Robust Implementation and Sustainability Model (PRISM). MDM Policy Pract.

[73] Glasgow RE, Studts T, Ford B (2022). RE-AIM online.

[74] Glasgow RE, Estabrooks PA, Ory MG (2020). Characterizing evolving frameworks: issues from Esmail et al. (2020) review. Implement Sci.

[75] Glasgow RE, Riley WT (2013). Pragmatic measures: what they are and why we need them. Am J Prev Med.

[76] Lewis CC, Mettert KD, Stanick CF, Halko HM, Nolen EA, Powell BJ (2021). The psychometric and pragmatic evidence rating scale (PAPERS) for measure development and evaluation. Implement Res Pract.

