# Supplementary material for: A citation analysis and scoping systematic review of the operationalization of the Practical, Robust Implementation and Sustainability Model (PRISM)
Source: Implement Sci. 2022 Sep 24;17:62. doi: 10.1186/s13012-022-01234-3 (PMC9509575; doi:10.1186/s13012-022-01234-3)
Supplement: Supplementary file 1 — Additional file 1. Abstraction form. [file 13012_2022_1234_MOESM1_ESM.pdf]

Abstraction Form for a Systematic Review of Practical, Robust Implementation and Sustainability Model (PRISM)

Primary Topic Area(s)

____
(Select up to 3)

If other, please specify

____

Study Setting

☐ Community
☐ School
☐ Faith-based
☐ Workplace
☐ Health Department (city, county, or state)
☐ National Health Initiative
☐ Clinical: In-Patient
☐ Clinical: Out-Patient
☐ Other

Other Study Setting

____

Study Location

☐ US
☐ Canada
☐ Europe
☐ Africa
☐ Central and South America
☐ Asia
☐ Australia/ NewZealand/ Nearby Islands
☐ Other

Name of Country (or Countries)

____

Was the study conducted in a low-middle income country?

○ Yes
○ No

Purpose Context

☐ Planning and development
☐ Implementation
☐ Evaluation
☐ Dissemination
☐ Sustainment
☐ Other

If other, please specify

____

Study Design	☐ RCT<br>☐ Cohort<br>☐ Case Control<br>☐ Observational<br>☐ Meta-Analysis<br>☐ Narrative Piece<br>☐ Pre/Post<br>☐ Case Study<br>☐ Stand-Alone Protocol Paper<br>☐ Other
If other, please specify	__________________________
Please search to see if this study team has since published more articles on this study.<br><br>Did you find more articles?	◯ Yes<br>◯ No
Paper 1 Citation	__________________________
Upload Paper 1 if available	
Paper 2 Citation	__________________________
Upload Paper 2 if available	
Paper 3 Citation	__________________________
Upload Paper 3 if available	
Study Type	☐ Efficacy<br>☐ Effectiveness<br>☐ Implementation<br>☐ HEI Type 1<br>☐ HEI Type 2<br>☐ HEI Type 3<br>☐ Dissemination<br>☐ Methods development<br>☐ Pilot study<br>☐ Other
If other, please specify	__________________________
Target Population	☐ Patient/community member level<br>☐ Provider/delivery agent level<br>☐ Setting level<br>☐ Other
If "other" target population, please describe.	__________________________

Patient/Community Sample Size

____
(NR= Not Reported)

Patient/Community population details

____

Provider Sample Size
(Please include unit. Ex. 24 physicians)

____
(NR= Not Reported)

Provider population details

____

Setting Sample Size
(Please include unit. Ex. 3 hospitals)

____
(NR= Not Reported)

Setting population details

____

Level/Unit of Study
(What level is the study randomizing at?)

- ◯ Individual (patient/participant)
- ◯ Recipient (provider/implementation staff)
- ◯ Setting
- ◯ Community
- ◯ Multi-Level (e.g. nested RCT)
- ◯ Other

If other, please specify

____

Methods Used

- ◯ Quantitative
- ◯ Qualitative
- ◯ Mixed Methods (only if quant/qual integrated)
- ◯ Narrative
- ◯ Multi-Methods (if quant/qual not integrated)
- ◯ Other

If other, please specify

____

Study objectives
(Copy from introduction or summarize in 2 sentences)

____

Please briefly describe the intervention 2-3 sentences

____

Please briefly describe the implementation strategy(ies) 2-3 sentences

____

Study Conclusions/Findings

____

Lessons learned (positive and negative)

____

General Study: Further Abstraction or Additional Notes

____

PRISM Use in Study

Study Name: [reviewer_1_arm_1][title_1]
First Author: [reviewer_1_arm_1][author1name_1]
Year of Publication: [reviewer_1_arm_1][year_1]
Abstract: [reviewer_1_arm_1][abstract_1]

PRISM use for this study

- ☐ Planning/Development
- ☐ Implementation
- ☐ Evaluation
- ☐ Dissemination
- ☐ Sustainment

Please rate the use/operationalization of PRISM domains for Planning
0-5; 0= Poor and 5= Excellent

____

Please explain score for use/operationalization for Planning

____

Please rate the use/operationalization of PRISM domains for Dissemination
0-5; 0= Poor and 5= Excellent

____

Please explain score for use/operationalization for Dissemination

____

Please rate the use/operationalization of PRISM domains for Implementation
0-5; 0= Poor and 5= Excellent

____

Please explain score for use/operationalization for Implementation

____

Please rate the use/operationalization of PRISM domains for Evaluation
0-5; 0= Poor and 5= Excellent

____

Please explain score for use/operationalization for Evaluation

____

Please rate the use/operationalization of PRISM domains for Sustainment
0-5; 0= Poor and 5= Excellent

____

Please explain score for use/operationalization for Sustainment

____

Was PRISM used with another framework?

◯ Yes
◯ No

What other framework(s) were used with PRISM?

____

Did the paper discuss adaptations to PRISM

◯ Yes
◯ No

Please describe how PRISM was adapted for this study.

____

Which PRISM domains were addressed in this study?

☐ Program/Intervention- Organizational
☐ Program/Intervention- Patient
☐ Recipients- Organizational Characteristics
☐ Recipients- Patient Characteristics
☐ Implementation and sustainability Infrastructure
☐ External Environment
☐ Did not report

PRISM Program/Intervention: Organizational Perspective

Which subdomains were assessed?

☐ Readiness
☐ Strength of the evidence base
☐ Addresses barriers of frontline staff
☐ Coordination across departments and specialties
☐ Usability and adaptability
☐ Trialability and reversibility
☐ Ability to observe results
☐ Burden (complexity and cost)
☐ Other: write in

Other PRISM Subdomain 1

____

Other PRISM Subdomain 2

____

Other PRISM Subdomain 3

____

How was this domain operationalized?
- ◯ Quantitative
- ◯ Qualitative
- ◯ Mixed Methods
- ◯ Narrative
- ◯ Multi-Methods
- ◯ Other

If other, please specify

____

RE-AIM Outcomes for this domain
- ☐ Reach
- ☐ Effectiveness
- ☐ Adoption: Setting
- ☐ Adoption: Staff
- ☐ Implementation
- ☐ Maintenance Individual
- ☐ Maintenance Organizational

How was this domain operationalized?
(Add information about instrument used, item, etc.)

____

PRISM Program/Intervention: Patient Perspective

Which subdomains were assessed?
- ☐ Patient centeredness
- ☐ Provides patient choices
- ☐ Addresses patient barriers
- ☐ Seamlessness of transition between program elements
- ☐ Feedback of results
- ☐ Service and access
- ☐ Burden (complexity and cost)
- ☐ Other: write in
- ☐

Other PRISM Subdomain 1

____

Other PRISM Subdomain 2

____

Other PRISM Subdomain 3

____

PRISM Use for this domain
- ◯ Quantitative
- ◯ Qualitative
- ◯ Mixed Methods
- ◯ Narrative
- ◯ Multi-Methods
- ◯ Other

How was this domain operationalized?

____

RE-AIM Outcomes for this domain	☐ Reach<br>☐ Effectiveness<br>☐ Adoption: Setting<br>☐ Adoption: Staff<br>☐ Implementation<br>☐ Maintenance Individual<br>☐ Maintenance Organizational
How was this domain operationalized?<br>(Add information about instrument used, item, etc.)	__________________________________

PRISM Recipients: Organizational Characteristics

Which subdomains were assessed?	☐ Organizational health and culture<br>☐ Management support and communication<br>☐ Clinical leadership<br>☐ Systems and training<br>☐ Data and decision support<br>☐ Staffing and incentives<br>☐ Expectation of sustainability<br>☐ Shared goals and cooperation<br>☐ Other: write in
Other PRISM Subdomain 1	__________________________________
Other PRISM Subdomain 2	__________________________________
Other PRISM Subdomain 3	__________________________________
PRISM Use for this domain	◯ Quantitative<br>◯ Qualitative<br>◯ Mixed Methods<br>◯ Narrative<br>◯ Multi-Methods<br>◯ Other
How was this domain operationalized?	__________________________________
RE-AIM Outcomes for this domain	☐ Reach<br>☐ Effectiveness<br>☐ Adoption: Setting<br>☐ Adoption: Staff<br>☐ Implementation<br>☐ Maintenance Individual<br>☐ Maintenance Organizational
How was this domain operationalized?<br>(Add information about instrument used, item, etc.)	__________________________________

PRISM Recipients: Patient Characteristics

Which subdomains were assessed?	☐ Demographics<br>☐ Disease burden<br>☐ Competing demands<br>☐ Knowledge and beliefs<br>☐ Other: write in
Other PRISM Subdomain 1	__________________________________
Other PRISM Subdomain 2	__________________________________
Other PRISM Subdomain 3	__________________________________
PRISM Use for this domain	◯ Quantitative<br>◯ Qualitative<br>◯ Mixed Methods<br>◯ Narrative<br>◯ Multi-Methods<br>◯ Other
How was this domain operationalized?	__________________________________
RE-AIM Outcomes for this domain	☐ Reach<br>☐ Effectiveness<br>☐ Adoption: Setting<br>☐ Adoption: Staff<br>☐ Implementation<br>☐ Maintenance Individual<br>☐ Maintenance Organizational
How was this domain operationalized?<br>(Add information about instrument used, item, etc.)	__________________________________

PRISM Implementation and Sustainability Infrastructure

Which subdomains were assessed?	☐ Performance data<br>☐ Dedicated team<br>☐ Adopter training and support<br>☐ Relationship and communication with adopters (bridge researchers)<br>☐ Facilitation of sharing of best practices<br>☐ Plan for sustainability<br>☐ Adaptable protocols and procedures<br>☐ Other: write in
Other PRISM Subdomain 1	__________________________________

Other PRISM Subdomain 2 | __________________

Other PRISM Subdomain 3 | __________________

PRISM Use for this domain

- ◯ Quantitative
- ◯ Qualitative
- ◯ Mixed Methods
- ◯ Narrative
- ◯ Multi-Methods
- ◯ Other

How was this domain operationalized? | __________________

RE-AIM Outcomes for this domain

- ☐ Reach
- ☐ Effectiveness
- ☐ Adoption: Setting
- ☐ Adoption: Staff
- ☐ Implementation
- ☐ Maintenance Individual
- ☐ Maintenance Organizational

How was this domain operationalized?
(Add information about instrument used, item, etc.) | __________________

PRISM External Environment

Which subdomains were assessed?

- ☐ Payor satisfaction
- ☐ Competition
- ☐ Regulatory environment
- ☐ Community resources
- ☐ Reimbursement
- ☐ Other: write in

Other PRISM Subdomain 1 | __________________

Other PRISM Subdomain 2 | __________________

Other PRISM Subdomain 3 | __________________

PRISM Use for this domain

- ◯ Quantitative
- ◯ Qualitative
- ◯ Mixed Methods
- ◯ Narrative
- ◯ Multi-Methods
- ◯ Other

How was this domain operationalized?

____

RE-AIM Outcomes for this domain

- ☐ Reach
- ☐ Effectiveness
- ☐ Adoption: Setting
- ☐ Adoption: Staff
- ☐ Implementation
- ☐ Maintenance Individual
- ☐ Maintenance Organizational

How was this domain operationalized?
(Add information about instrument used, item, etc.)

____

PRISM Miscellaneous comments/information

Please use this space for any additional notes/comments

____

RE-AIM Outcomes at the Study Level

RE-AIM use for this study

- ☐ Planning
- ☐ Implementation
- ☐ Evaluation
- ☐ Dissemination
- ☐ Sustainment
- ☐ Not Reported

Please rate the use/operationalization of RE-AIM for Planning
0-5; 0= Poor and 5= Excellent

____

Please explain score for use/operationalization for Planning

____

Please rate the use/operationalization of RE-AIM for Dissemination
0-5; 0= Poor and 5= Excellent

____

Please explain score for use/operationalization for Dissemination

____

Please rate the use/operationalization of RE-AIM for Implementation
0-5; 0= Poor and 5= Excellent

____

Please explain score for use/operationalization for Implementation

____

Please rate the use/operationalization of RE-AIM for Evaluation<br>0-5; 0= Poor and 5= Excellent	__________________________________
Please explain score for use/operationalization for Evaluation	__________________________________
Please rate the use/operationalization of RE-AIM for Sustainment<br>0-5; 0= Poor and 5= Excellent	__________________________________
Please explain score for use/operationalization for Sustainment	__________________________________
Please select the RE-AIM outcomes for this study	☐ Reach<br>☐ Effectiveness<br>☐ Adoption: Setting<br>☐ Adoption: Staff<br>☐ Implementation<br>☐ Maintenance Individual<br>☐ Maintenance Organizational<br>☐ Did not report

RE-AIM Reach

How was Reach operationalized for this study?	◯ Quantitative<br>◯ Qualitative<br>◯ Mixed Methods<br>◯ Narrative<br>◯ Multi-Methods<br>◯ Other
If other, please specify	__________________________________
How was Reach operationalized for this study?	__________________________________
Findings for Reach	__________________________________

RE-AIM Effectiveness

How was Effectiveness operationalized for this study?	◯ Quantitative<br>◯ Qualitative<br>◯ Mixed Methods<br>◯ Narrative<br>◯ Multi-Methods<br>◯ Other
If other, please specify	__________________________________

How was Effectiveness operationalized for this study?

____

Findings for Effectiveness

____

RE-AIM Adoption: Setting

How was Adoption in the Setting operationalized for this study?

- ◯ Quantitative
- ◯ Qualitative
- ◯ Mixed Methods
- ◯ Narrative
- ◯ Multi-Methods
- ◯ Other

If other, please specify

____

How was Adoption in the Setting operationalized for this study?

____

Findings for Adoption in the Setting

____

RE-AIM Adoption: Staff

How was Adoption for the Staff operationalized for this study?

- ◯ Quantitative
- ◯ Qualitative
- ◯ Mixed Methods
- ◯ Narrative
- ◯ Multi-Methods
- ◯ Other

If other, please specify

____

How was Adoption for the Staff operationalized for this study?

____

Findings for Adoption for the Staff

____

RE-AIM Implementation

How was Implementation operationalized for this study?

- ◯ Quantitative
- ◯ Qualitative
- ◯ Mixed Methods
- ◯ Narrative
- ◯ Multi-Methods
- ◯ Other

If other, please specify

____

How was Implementation operationalized for this study?

____

Findings for Implementation

____

RE-AIM Maintenance Individual

How was Maintenance for the Individual operationalized for this study?

- ◯ Quantitative
- ◯ Qualitative
- ◯ Mixed Methods
- ◯ Narrative
- ◯ Multi-Methods
- ◯ Other

If other, please specify

____

How was Maintenance for the Individual operationalized for this study?

____

Findings for Maintenance for the Individual

____

RE-AIM Maintenance Organizational

How was Maintenance for the Organization operationalized for this study?

- ◯ Quantitative
- ◯ Qualitative
- ◯ Mixed Methods
- ◯ Narrative
- ◯ Multi-Methods
- ◯ Other

If other, please specify

____

How was Maintenance for the Organization operationalized for this study?

____

Findings for Maintenance for the Organization

____

RE-AIM Miscellaneous comments/information

Please use this space for any additional notes/comments

____
