# Supplementary material for: A citation analysis and scoping systematic review of the operationalization of the Practical, Robust Implementation and Sustainability Model (PRISM)
Source: Implement Sci. 2022 Sep 24;17:62. doi: 10.1186/s13012-022-01234-3 (PMC9509575; doi:10.1186/s13012-022-01234-3)
Supplement: Supplementary file 2 — Additional file 2. All papers included in the Practical, Robust Implementation and Sustainability Model review with level of use and search source. [file 13012_2022_1234_MOESM2_ESM.docx]

Additional file 2: All papers included in the Practical, Robust Implementation and Sustainability Model review with level of use and search source

| **Citation** | **Search Source** |
| --- | --- |
| **Integrated PRISM** | |
| Ameling, et al., 2014^1^ | Reverse |
| Ayele, et al., 2017^2^ | Reverse |
| Ayele, et al., 2019^3^ | Reverse |
| Beck, et al., 2009^4^ | Reverse |
| Ekawati, et al., 2019^5^ | Reverse |
| Ekawati, et al., 2020^6^ | Abstraction |
| Esses, et al., 2019^7^ | PubMed |
| Feldstein, et al., 2008^8^ | Reverse |
| Gopalan, et al., 2014^9^ | Reverse |
| Gopalan, et al., 2016^10^ | Reverse |
| Gopalan, et al., 2019^11^ | Reverse |
| Henderson, et al., 2020^12^ | PubMed |
| Knudsen, et al., 2020^13^ | Recommended |
| Leonard, et al., 2017^14^ | Reverse |
| Leonard, et al., 2019^15^ | Reverse |
| Li, et al., 2020^16^ | Reverse |
| Liles, et al., 2015^17^ | Reverse |
| Linke, et al., 2020^18^ | Recommended |
| McCreight, et al., 2019^19^ | PubMed |
| McKay, et al., 2020^20^ | Reverse |
| Paniagua-Avila, et al., 2020^21^ | PubMed |
| Pittman, et al., 2020^22^ | PubMed |
| Satre, et al., 2019^23^ | Reverse |
| Schneider, et al., 2016^24^ | Reverse |
| Schölin, et al., 2019^25^ | Reverse |
| Shields, et al., 2020^26^ | PubMed |
| Ssewamala, et al., 2018^27^ | Reverse |
| Stephens, et al., 2014^28^ | Reverse |
| Sullivan, et al., 2018^29^ | PubMed |
| Woodbridge, et al., 2014^30^ | Web Of Science |
| Yakovchenko, et al., 2019^31^ | Reverse |
| Zhang, et al., 2020^32^ | Reverse |
| **Directed by PRISM** | |
| Alostad · 2019^33^ | Reverse |
| Aquilante, et al., 2020^34^ | Reverse |
| Blok · 2019^35^ | Reverse |
| Chang, et al., 2020^36^ | Recommended |
| DeBar · 2018^37^ | Reverse |
| Fathauer, et al., 2012^38^ | Reverse |
| Feldstein, et al., 2008^39^ | Reverse |
| Gilmartin · 2019^40^ | Reverse |
| Jones · 2020^41^ | Reverse |
| Karki · 2018^42^ | Reverse |
| Liddy · 2016^43^ | Reverse |
| Liddy · 2018^44^ | Reverse |
| Liu · 2020^45^ | Reverse |
| Magee · 2019^46^ | Reverse |
| McMullen · 2019^47^ | Reverse |
| Meyerson · 2015^48^ | Reverse |
| Miller · 2019^49^ | Reverse |
| Napolitano · 2017^50^ | Reverse |
| Pedersen · 2018^51^ | Reverse |
| Smith · 2012^52^ | Reverse |
| Trinkley, et al., 2020^53^ | Recommended |
| Warriner · 2014^54^ | Reverse |
| Zhong · 2015^55^ | Reverse |
| **Informed by PRISM** | |
| Chamberlain · 2012^56^ | Reverse |
| Chuang · 2015^57^ | Reverse |
| Damschroder · 2009^58^ | Reverse |
| Feil · 2014^59^ | Reverse |
| Irvine · 2013^60^ | Reverse |
| Kind · 2016^61^ | Reverse |
| Meissner · 2018^62^ | Reverse |
| Munthe-Kaas · 2019^63^ | Reverse |
| Naidoo · 2018^64^ | Reverse |
| Stirman · 2012^65^ | Reverse |
| Woodcock · 2020^66^ | Reverse |
| **Framing the Field** | |
| Bearman · 2014^67^ | Reverse |
| Burnham, 2020^68^ | Reverse |
| DeBar · 2012^69^ | Reverse |
| Demiris · 2014^70^ | Reverse |
| Glasgow · 2019^71^ | Reverse |
| Gopalan · 2020^72^ | Reverse |
| Gupta · 2017^73^ | Reverse |
| Hanson · 2016^74^ | Reverse |
| Harden · 2018^75^ | Reverse |
| Harden · 2020^76^ | Reverse |
| Hodge · 2017^77^ | Reverse |
| Holt · 2017^78^ | Reverse |
| Holtrop · 2018^79^ | Reverse |
| Hudson · 2009^80^ | Reverse |
| Kwan · 2019^81^ | Reverse |
| Lewis · 2017^82^ | Reverse |
| Magee · 2017^83^ | Reverse |
| Manojlovich · 2015^84^ | Reverse |
| Matlock, et al., 2020^85^ | Recommended |
| Mayer · 2018^86^ | Reverse |
| Mogaka, et al., 2020^87^ | Recommended |
| Quinn · 2019^88^ | Reverse |
| Schloemer · 2018^89^ | Reverse |
| Shelton · 2020^90^ | Reverse |
| Shelton · 2020 ^91^ | Reverse |
| Stange · 2012^92^ | Reverse |
| Tan · 2018^93^ | Reverse |
| Weiss · 2016^94^ | Reverse |
| Yu, 2015^95^ | Reverse |
| **Review** | |
| Beauchemin · 2019^96^ | Reverse |
| Cambon · 2012^97^ | Reverse |
| Davison · 2015^98^ | Reverse |
| Dintrans · 2019^99^ | Reverse |
| Gagnon · 2014^100^ | Reverse |
| Harris · 2017^101^ | Reverse |
| Lennox · 2018^102^ | Reverse |
| Mitchell · 2010^103^ | Reverse |
| Moullin · 2015^104^ | Reverse |
| Nilsen · 2019^105^ | Reverse |
| Skolarus · 2017^106^ | Reverse |
| Tabak · 2012^107^ | Reverse |
| Wilson · 2010^108^ | Reverse |
| Wisdom · 2014^109^ | Reverse |
| **Referenced PRISM** |  |
| Aarons · 2011^110^ | Reverse |
| Allen, et al., 2020^111^ | Recommended |
| Andrews · 2014^112^ | Reverse |
| Bayliss · 2014^113^ | Reverse |
| Becker · 2011^114^ | Reverse |
| Beehler · 2013^115^ | Reverse |
| Bradley · 2017^116^ | Reverse |
| Broder-Fingert · 2018^117^ | Reverse |
| Cambon · 2013^118^ | Reverse |
| Candas · 2016^119^ | Reverse |
| Chamberlain · 2011^120^ | Reverse |
| Chaudoir · 2013^121^ | Reverse |
| Chor · 2015^122^ | Reverse |
| Colldén · 2018^123^ | Reverse |
| Damian · 2017^124^ | Reverse |
| Faro · 2019^125^ | Reverse |
| Fenwick · 2020^126^ | Reverse |
| Fitzgerald · 2015^127^ | Reverse |
| Garland · 2013^128^ | Reverse |
| Gitlin · 2015^129^ | Reverse |
| Goodman · 2017^130^ | Reverse |
| Hartveit · 2019^131^ | Reverse |
| Herlitz · 2020^132^ | Reverse |
| Hurlburt · 2014^133^ | Reverse |
| Iliffe · 2008^134^ | Reverse |
| Johnson · 2014^135^ | Reverse |
| King · 2020^136^ | Reverse |
| Kolko · 2010^137^ | Reverse |
| Kuiper · 2015^138^ | Reverse |
| LaMonica · 2019^139^ | Reverse |
| Leeman · 2019^140^ | Reverse |
| Lengnick-Hall, et al., 2020^141^ | Reverse |
| Lennox · 2017^142^ | Reverse |
| Lewis · 2016^143^ | Reverse |
| Lloyd · 2020^144^ | Reverse |
| Martinez · 2014^145^ | Reverse |
| McAlearney · 2016^146^ | Reverse |
| McCullough · 2015^147^ | Reverse |
| Morrato · 2015^148^ | Reverse |
| Munoz-Plaza · 2016^149^ | Reverse |
| Nadeem · 2011^150^ | Reverse |
| Nadeem · 2013^151^ | Reverse |
| Nadeem · 2013^152^ | Reverse |
| Nadeem · 2013^153^ | Reverse |
| Nadeem · 2014^154^ | Reverse |
| O’Malley · 2014^155^ | Reverse |
| Okere · 2020^156^ | Reverse |
| Olin · 2015^157^ | Reverse |
| Olin · 2016^158^ | Reverse |
| Pannebakker · 2019^159^ | Reverse |
| Pednekar · 2018^160^ | Reverse |
| Pescheny · 2018^161^ | Reverse |
| Prendergast · 2017^162^ | Reverse |
| Proctor · 2012^163^ | Reverse |
| Ramsey · 2019^164^ | Reverse |
| Ratcliff · 2019^165^ | Reverse |
| Robins · 2013^166^ | Reverse |
| Rongey · 2011^167^ | Reverse |
| Rotteau · 2015^168^ | Reverse |
| Stirman · 2017^169^ | Reverse |
| Stirman · 2018^170^ | Reverse |
| Sullivan · 2018^171^ | Reverse |
| Sullivan · 2018^172^ | Reverse |
| Swinkels · 2018^173^ | Reverse |
| Teri · 2012^174^ | Reverse |
| Trompette · 2014^175^ | Reverse |
| Urquhart · 2019^176^ | Reverse |
| Van Acker · 2012^177^ | Reverse |
| Ward · 2017^178^ | Reverse |
| Weaver · 2014^179^ | Reverse |
| Weaver · 2019^180^ | Reverse |
| Welsh · 2016^181^ | Reverse |

1. Ameling JM, Ephraim PL, Bone LR, et al. Adapting hypertension self-management interventions to enhance their sustained effectiveness among urban African Americans. *Fam Community Health.* 2014;37(2):119-133.

2. Ayele RA, Lawrence E, McCreight M, et al. Study protocol: improving the transition of care from a non-network hospital back to the patient's medical home. *BMC health services research.* 2017;17(1):123.

3. Ayele RA, Lawrence E, McCreight M, et al. Perspectives of Clinicians, Staff, and Veterans in Transitioning Veterans from non-VA Hospitals to Primary Care in a Single VA Healthcare System. *J Hosp Med.* 2019;14(3):E1-e7.

4. Beck A, Bergman DA, Rahm AK, Dearing JW, Glasgow RE. Using Implementation and Dissemination Concepts to Spread 21st-century Well-Child Care at a Health Maintenance Organization. *Perm J.* 2009;13(3):10-18.

5. Ekawati FM, Licqurish S, Emilia O, Gunn J, Brennecke S, Lau P. Developing management pathways for hypertensive disorders of pregnancy (HDP) in Indonesian primary care: a study protocol. *Reprod Health.* 2019;16(1):12.

6. Ekawati FM, Emilia O, Gunn J, Licqurish S, Lau P. The elephant in the room: an exploratory study of hypertensive disorders of pregnancy (HDP) management in Indonesian primary care settings. *BMC Family Practice.* 2020;21(1):242.

7. Esses SA, Small S, Rodemann A, Hartman ME. Post-Intensive Care Syndrome: Educational Interventions for Parents of Hospitalized Children. *Am J Crit Care.* 2019;28(1):19-27.

8. Feldstein AC, Glasgow RE. A practical, robust implementation and sustainability model (PRISM) for integrating research findings into practice. *Jt Comm J Qual Patient Saf.* 2008;34(4):228-243.

9. Gopalan G, Franco LM, Dean-Assael K, McGuire-Schwartz M, Chacko A, McKay M. Statewide implementation of the 4 Rs and 2 Ss for strengthening families. *J Evid Based Soc Work.* 2014;11(1-2):84-96.

10. Gopalan G. Feasibility of improving child behavioral health using task-shifting to implement the 4Rs and 2Ss program for strengthening families in child welfare. *Pilot Feasibility Stud.* 2016;2.

11. Gopalan G, Hooley C, Winters A, Stephens T. Perceptions Among Child Welfare Staff when Modifying A Child Mental Health Intervention to be Implemented in Child Welfare Services. *Am J Community Psychol.* 2019;63(3-4):366-377.

12. Henderson V, Tossas-Milligan K, Martinez E, et al. Implementation of an integrated framework for a breast cancer screening and navigation program for women from underresourced communities. *Cancer.* 2020;126 Suppl 10:2481-2493.

13. Knudsen HK, Drainoni M-L, Gilbert L, et al. Model and approach for assessing implementation context and fidelity in the HEALing Communities Study. *Drug and Alcohol Dependence.* 2020;217:108330.

14. Leonard C, Lawrence E, McCreight M, et al. Implementation and dissemination of a transition of care program for rural veterans: a controlled before and after study. *Implement Sci.* 2017;12(1):123.

15. Leonard C, Gilmartin H, McCreight M, et al. Operationalizing an Implementation Framework to Disseminate a Care Coordination Program for Rural Veterans. *J Gen Intern Med.* 2019;34(Suppl 1):58-66.

16. Li L, Zhang R, Chen Y, et al. Achievements and challenges in health management for aged individuals in primary health care sectors: a survey in Southwest China. *BMC public health.* 2020;20(1):338.

17. Liles EG, Schneider JL, Feldstein AC, et al. Implementation challenges and successes of a population-based colorectal cancer screening program: a qualitative study of stakeholder perspectives. *Implement Sci.* 2015;10:41.

18. Linke SE, Kallenberg GR, Kronick R, Tai-Seale M, De-Guzman K, Rabin B. Integrating “Exercise Is Medicine” into primary care workflow: a study protocol. *Translational behavioral medicine.* 2020.

19. McCreight MS, Rabin BA, Glasgow RE, et al. Using the Practical, Robust Implementation and Sustainability Model (PRISM) to qualitatively assess multilevel contextual factors to help plan, implement, evaluate, and disseminate health services programs. *Translational behavioral medicine.* 2019;9(6):1002-1011.

20. McKay MM, Sensoy Bahar O, Ssewamala FM. Implementation science in global health settings: Collaborating with governmental & community partners in Uganda. *Psychiatry Res.* 2020;283:112585.

21. Paniagua-Avila A, Fort MP, Glasgow RE, et al. Evaluating a multicomponent program to improve hypertension control in Guatemala: study protocol for an effectiveness-implementation cluster randomized trial. *Trials.* 2020;21(1):509.

22. Pittman JOE, Davidson EJ, Dozier ME, et al. Implementation and evaluation of a community-based treatment for late-life hoarding. *Int Psychogeriatr.* 2020:1-10.

23. Satre DD, Anderson AN, Leibowitz AS, et al. Implementing electronic substance use disorder and depression and anxiety screening and behavioral interventions in primary care clinics serving people with HIV: Protocol for the Promoting Access to Care Engagement (PACE) trial. *Contemp Clin Trials.* 2019;84:105833.

24. Schneider JL, Davis J, Kauffman TL, et al. Stakeholder perspectives on implementing a universal Lynch syndrome screening program: a qualitative study of early barriers and facilitators. *Genet Med.* 2016;18(2):152-161.

25. Schölin L, Fitzgerald N. The conversation matters: a qualitative study exploring the implementation of alcohol screening and brief interventions in antenatal care in Scotland. *BMC Pregnancy Childbirth.* 2019;19(1):316.

26. Shields N, Willis C, Imms C, et al. FitSkills: protocol for a stepped wedge cluster randomised trial of a community-based exercise programme to increase participation among young people with disability. *BMJ Open.* 2020;10(7):e037153.

27. Ssewamala FM, Sensoy Bahar O, McKay MM, Hoagwood K, Huang KY, Pringle B. Strengthening mental health and research training in Sub-Saharan Africa (SMART Africa): Uganda study protocol. *Trials.* 2018;19(1):423.

28. Stephens TN, McGuire-Schwartz M, Rotko L, Fuss A, McKay MM. A learning collaborative supporting the implementation of an evidence-informed program, the "4Rs and 2Ss for children with conduct difficulties and their families". *J Evid Based Soc Work.* 2014;11(5):511-523.

29. Sullivan JL, Shin MH, Engle RL, et al. Evaluating the Implementation of Project Re-Engineered Discharge (RED) in Five Veterans Health Administration (VHA) Hospitals. *Jt Comm J Qual Patient Saf.* 2018;44(11):663-673.

30. Woodbridge MW, Sumi WC, Yu J, et al. Implementation and Sustainability of an Evidence-Based Program Lessons Learned From the PRISM Applied to First Step to Success. *J Emot Behav Disord.* 2014;22(2):95-106.

31. Yakovchenko V, Hogan TP, Houston TK, et al. Automated text messaging with patients in Department of Veterans Affairs specialty clinics: Cluster randomized trial. *J Med Internet Res.* 2019;21(8).

32. Zhang R, Chen Y, Liu S, et al. Progress of equalizing basic public health services in Southwest China--- health education delivery in primary healthcare sectors. *BMC health services research.* 2020;20(1):247.

33. Alostad AH, Steinke DT, Schafheutle EI. A qualitative exploration of Bahrain and Kuwait herbal medicine registration systems: policy implementation and readiness to change. *J Pharm Policy Pract.* 2019;12:32.

34. Aquilante CL, Kao DP, Trinkley KE, et al. Clinical implementation of pharmacogenomics via a health system-wide research biobank: the University of Colorado experience. *Pharmacogenomics.* 2020;21(6):375-386.

35. Blok AC, Sadasivam RS, Hogan TP, Patterson A, Day N, Houston TK. Nurse-Driven mHealth Implementation Using the Technology Inpatient Program for Smokers (TIPS): Mixed Methods Study. *JMIR Mhealth Uhealth.* 2019;7(10):e14331.

36. Chang ET, Oberman RS, Cohen AN, et al. Increasing Access to Medications for Opioid Use Disorder and Complementary and Integrative Health Services in Primary Care. *Journal of General Internal Medicine.* 2020.

37. DeBar L, Benes L, Bonifay A, et al. Interdisciplinary team-based care for patients with chronic pain on long-term opioid treatment in primary care (PPACT) - Protocol for a pragmatic cluster randomized trial. *Contemp Clin Trials.* 2018;67:91-99.

38. Fathauer L, Meek J. Initial implementation and evaluation of a Hepatitis C treatment clinical decision support system (CDSS): a nurse practitioner-driven quality improvement initiative. *Appl Clin Inform.* 2012;3(3):337-348.

39. Feldstein AC, Schneider J, Smith DH, et al. Harnessing stakeholder perspectives to improve the care of osteoporosis after a fracture. *Osteoporos Int.* 2008;19(11):1527-1540.

40. Gilmartin H, Lawrence E, Leonard C, et al. Brainwriting Premortem: A Novel Focus Group Method to Engage Stakeholders and Identify Preimplementation Barriers. *J Nurs Care Qual.* 2019;34(2):94-100.

41. Jones LK, Gidding SS, Seaton TL, et al. Developing implementation strategies to improve uptake of guideline-recommended treatments for individuals with familial hypercholesterolemia: A protocol. *Res Social Adm Pharm.* 2020;16(3):390-395.

42. Karki P, Prabandari YS, Probandari A, Banjara MR. Feasibility of school-based health education intervention to improve the compliance to mass drug administration for lymphatic Filariasis in Lalitpur district, Nepal: A mixed methods among students, teachers and health program manager. *PLoS One.* 2018;13(9):e0203547.

43. Liddy C, Rowan M, Valiquette-Tessier SC, Drosinis P, Crowe L, Hogg W. Improved Delivery of Cardiovascular Care (IDOCC): Findings from Narrative Reports by Practice Facilitators. *Prev Med Rep.* 2016;5:214-219.

44. Liddy C, Rowan M, Valiquette-Tessier SC, Drosinis P, Crowe L, Hogg W. Experiences of practice facilitators working on the Improved Delivery of Cardiovascular Care project: Retrospective case study. *Can Fam Physician.* 2018;64(1):e23-e32.

45. Liu Y, Wu X, Cai C, et al. Peer support in Shanghai's Commitment to diabetes and chronic disease self-management: program development, program expansion, and policy. *Translational behavioral medicine.* 2020;10(1):13-24.

46. Magee MF, Baker KM, Fernandez SJ, et al. Redesigning ambulatory care management for uncontrolled type 2 diabetes: a prospective cohort study of the impact of a Boot Camp model on outcomes. *BMJ Open Diabetes Res Care.* 2019;7(1):e000731.

47. McMullen CK, Rosetti MO, Weinmann S, Leo MC, Nielsen ME. Clinical Use Cases for a Tool to Assess Risk in Superficial Bladder Cancer. *Perm J.* 2019;23.

48. Meyerson BE, Sayegh MA, Davis A, et al. Cervical cancer screening in a sexually transmitted disease clinic: screening adoption experiences from a midwestern clinic. *American journal of public health.* 2015;105 Suppl 2(Suppl 2):e8-14.

49. Miller LB, Sjoberg H, Mayberry A, McCreight MS, Ayele RA, Battaglia C. The advanced care coordination program: a protocol for improving transitions of care for dual-use veterans from community emergency departments back to the Veterans Health Administration (VA) primary care. *BMC health services research.* 2019;19(1):734.

50. Napolitano MA, Whiteley JA, Mavredes MN, et al. Using social media to deliver weight loss programming to young adults: Design and rationale for the Healthy Body Healthy U (HBHU) trial. *Contemp Clin Trials.* 2017;60:1-13.

51. Pedersen NH, Koch S, Larsen KT, et al. Protocol for evaluating the impact of a national school policy on physical activity levels in Danish children and adolescents: the PHASAR study - a natural experiment. *BMC public health.* 2018;18(1):1245.

52. Smith DH, Schneider J, Thorp ML, et al. Clinician's use of automated reports of estimated glomerular filtration rate: a qualitative study. *BMC Nephrol.* 2012;13:154.

53. Trinkley KE, Kahn MG, Bennett TD, et al. Integrating the Practical Robust Implementation and Sustainability Model With Best Practices in Clinical Decision Support Design: Implementation Science Approach. *J Med Internet Res.* 2020;22(10):e19676.

54. Warriner AH, Outman RC, Feldstein AC, et al. Effect of self-referral on bone mineral density testing and osteoporosis treatment. *Medical care.* 2014;52(8):743-750.

55. Zhong X, Wang Z, Fisher EB, Tanasugarn C. Peer Support for Diabetes Management in Primary Care and Community Settings in Anhui Province, China. *Ann Fam Med.* 2015;13 Suppl 1(Suppl 1):S50-58.

56. Chamberlain P, Roberts R, Jones H, Marsenich L, Sosna T, Price JM. Three collaborative models for scaling up evidence-based practices. *Adm Policy Ment Health.* 2012;39(4):278-290.

57. Chuang E, Ayala GX, Schmied E, Ganter C, Gittelsohn J, Davison KK. Evaluation protocol to assess an integrated framework for the implementation of the Childhood Obesity Research Demonstration project at the California (CA-CORD) and Massachusetts (MA-CORD) sites. *Child Obes.* 2015;11(1):48-57.

58. Damschroder LJ, Aron DC, Keith RE, Kirsh SR, Alexander JA, Lowery JC. Fostering implementation of health services research findings into practice: a consolidated framework for advancing implementation science. *Implement Sci.* 2009;4:50.

59. Feil EG, Frey A, Walker HM, et al. The Efficacy of a Home-School Intervention for Preschoolers With Challenging Behaviors: A Randomized Controlled Trial of Preschool First Step to Success. *J Early Interv.* 2014;36(3):151-170.

60. Irvine AB, Billow MB, McMahon E, Eberhage MG, Seeley JR, Bourgeois M. Mental illness training on the Internet for nurse aides: a replication study. *J Psychiatr Ment Health Nurs.* 2013;20(10):902-912.

61. Kind AJ, Brenny-Fitzpatrick M, Leahy-Gross K, et al. Harnessing Protocolized Adaptation in Dissemination: Successful Implementation and Sustainment of the Veterans Affairs Coordinated-Transitional Care Program in a Non-Veterans Affairs Hospital. *J Am Geriatr Soc.* 2016;64(2):409-416.

62. Meissner P. LADDERS: A dynamic paradigm for planning, implementing, and evaluating sustainable change in learning health systems. *Learn Health Syst.* 2018;2(3):e10058.

63. Munthe-Kaas H, Nøkleby H, Nguyen L. Systematic mapping of checklists for assessing transferability. *Syst Rev.* 2019;8(1):22.

64. Naidoo N, Zuma N, Khosa NS, et al. Qualitative assessment of facilitators and barriers to HIV programme implementation by community health workers in Mopani district, South Africa. *PLoS One.* 2018;13(8):e0203081.

65. Wiltsey Stirman S, Kimberly J, Cook N, Calloway A, Castro F, Charns M. The sustainability of new programs and innovations: a review of the empirical literature and recommendations for future research. *Implement Sci.* 2012;7:17.

66. Woodcock T, Adeleke Y, Goeschel C, Pronovost P, Dixon-Woods M. A modified Delphi study to identify the features of high quality measurement plans for healthcare improvement projects. *BMC Med Res Methodol.* 2020;20(1):8.

67. Bearman G, Stevens MP. Pushing beyond resistors and constipators: implementation considerations for infection prevention best practices. *Curr Infect Dis Rep.* 2014;16(1):388.

68. Burnham JP, Geng E, Venkatram C, Colditz GA, McKay VR. Putting the Dissemination and Implementation in Infectious Diseases. *Clin Infect Dis.* 2020;71(1):218-225.

69. Debar LL, Kindler L, Keefe FJ, et al. A primary care-based interdisciplinary team approach to the treatment of chronic pain utilizing a pragmatic clinical trials framework. *Translational behavioral medicine.* 2012;2(4):523-530.

70. Demiris G, Parker Oliver D, Capurro D, Wittenberg-Lyles E. Implementation science: implications for intervention research in hospice and palliative care. *Gerontologist.* 2014;54(2):163-171.

71. Glasgow RE, Harden SM, Gaglio B, et al. RE-AIM Planning and Evaluation Framework: Adapting to New Science and Practice With a 20-Year Review. *Front Public Health.* 2019;7:64.

72. Gopalan G, Bunger AC, Powell BJ. Skills for Developing and Maintaining Community-Partnerships for Dissemination and Implementation Research in Children's Behavioral Health: Implications for Research Infrastructure and Training of Early Career Investigators. *Adm Policy Ment Health.* 2020;47(2):227-243.

73. Gupta S, Nodora J. Optimizing the Quality of the Colorectal Cancer Screening Continuum: A Call to Action. *Journal of the National Cancer Institute.* 2017;109(5).

74. Hanson RF, Self-Brown S, Rostad WL, Jackson MC. The what, when, and why of implementation frameworks for evidence-based practices in child welfare and child mental health service systems. *Child Abuse Negl.* 2016;53:51-63.

75. Harden SM, Smith ML, Ory MG, Smith-Ray RL, Estabrooks PA, Glasgow RE. RE-AIM in Clinical, Community, and Corporate Settings: Perspectives, Strategies, and Recommendations to Enhance Public Health Impact. *Front Public Health.* 2018;6:71.

76. Harden SM, Strayer TE, 3rd, Smith ML, et al. National Working Group on the RE-AIM Planning and Evaluation Framework: Goals, Resources, and Future Directions. *Front Public Health.* 2019;7:390.

77. Hodge LM, Turner KMT, Sanders MR, Filus A. Sustained Implementation Support Scale: Validation of a Measure of Program Characteristics and Workplace Functioning for Sustained Program Implementation. *J Behav Health Serv Res.* 2017;44(3):442-464.

78. Holt CL, Chambers DA. Opportunities and challenges in conducting community-engaged dissemination/implementation research. *Translational behavioral medicine.* 2017;7(3):389-392.

79. Holtrop JS, Rabin BA, Glasgow RE. Qualitative approaches to use of the RE-AIM framework: rationale and methods. *BMC health services research.* 2018;18(1):177.

80. Hudson SV, Chubak J, Coups EJ, et al. Identifying key questions to advance research and practice in cancer survivorship follow-up care: a report from the ASPO Survivorship Interest Group. *Cancer epidemiology, biomarkers & prevention : a publication of the American Association for Cancer Research, cosponsored by the American Society of Preventive Oncology.* 2009;18(7):2152-2154.

81. Kwan BM, McGinnes HL, Ory MG, Estabrooks PA, Waxmonsky JA, Glasgow RE. RE-AIM in the Real World: Use of the RE-AIM Framework for Program Planning and Evaluation in Clinical and Community Settings. *Front Public Health.* 2019;7:345.

82. Lewis BA, Napolitano MA, Buman MP, Williams DM, Nigg CR. Future directions in physical activity intervention research: expanding our focus to sedentary behaviors, technology, and dissemination. *J Behav Med.* 2017;40(1):112-126.

83. Magee M, Bardsley JK, Wallia A, Smith KM. Transitioning the Adult with Type 2 Diabetes From the Acute to Chronic Care Setting: Strategies to Support Pragmatic Implementation Success. *Curr Diab Rep.* 2017;17(1):6.

84. Manojlovich M, Squires JE, Davies B, Graham ID. Hiding in plain sight: communication theory in implementation science. *Implement Sci.* 2015;10:58.

85. Matlock DD, Fukunaga MI, Tan A, et al. Enhancing Success of Medicare’s Shared Decision Making Mandates Using Implementation Science: Examples Applying the Pragmatic Robust Implementation and Sustainability Model (PRISM). *MDM Policy & Practice.* 2020;5(2):2381468320963070.

86. Mayer KH, Chan PA, R RP, Flash CA, Krakower DS. Evolving Models and Ongoing Challenges for HIV Preexposure Prophylaxis Implementation in the United States. *J Acquir Immune Defic Syndr.* 2018;77(2):119-127.

87. Mogaka JJO, James SE, Chimbari MJ. Leveraging implementation science to improve implementation outcomes in precision medicine. *Am J Transl Res.* 2020;12(9):4853-4872.

88. Quinn AK, Neta G, Sturke R, et al. Adapting and Operationalizing the RE-AIM Framework for Implementation Science in Environmental Health: Clean Fuel Cooking Programs in Low Resource Countries. *Front Public Health.* 2019;7:389.

89. Schloemer T, Schröder-Bäck P. Criteria for evaluating transferability of health interventions: a systematic review and thematic synthesis. *Implement Sci.* 2018;13(1):88.

90. Shelton RC, Chambers DA, Glasgow RE. An Extension of RE-AIM to Enhance Sustainability: Addressing Dynamic Context and Promoting Health Equity Over Time. *Front Public Health.* 2020;8:134.

91. Shelton RC, Lee M, Brotzman LE, Wolfenden L, Nathan N, Wainberg ML. What Is Dissemination and Implementation Science?: An Introduction and Opportunities to Advance Behavioral Medicine and Public Health Globally. *Int J Behav Med.* 2020;27(1):3-20.

92. Stange KC, Breslau ES, Dietrich AJ, Glasgow RE. State-of-the-art and future directions in multilevel interventions across the cancer control continuum. *J Natl Cancer Inst Monogr.* 2012;2012(44):20-31.

93. Tan ASL, Mazor KM, McDonald D, et al. Designing Shared Decision-Making Interventions for Dissemination and Sustainment: Can Implementation Science Help Translate Shared Decision Making Into Routine Practice? *MDM Policy Pract.* 2018;3(2):2381468318808503.

94. Weiss CH, Krishnan JA, Au DH, et al. An Official American Thoracic Society Research Statement: Implementation Science in Pulmonary, Critical Care, and Sleep Medicine. *Am J Respir Crit Care Med.* 2016;194(8):1015-1025.

95. Yu X, Klesges LM, Smeltzer MP, Osarogiagbon RU. Measuring improvement in populations: implementing and evaluating successful change in lung cancer care. *Transl Lung Cancer Res.* 2015;4(4):373-384.

96. Beauchemin M, Cohn E, Shelton RC. Implementation of Clinical Practice Guidelines in the Health Care Setting: A Concept Analysis. *ANS Adv Nurs Sci.* 2019;42(4):307-324.

97. Cambon L, Minary L, Ridde V, Alla F. Transferability of interventions in health education: a review. *BMC public health.* 2012;12:497.

98. Davison CM, Ndumbe-Eyoh S, Clement C. Critical examination of knowledge to action models and implications for promoting health equity. *International journal for equity in health.* 2015;14:49.

99. Villalobos Dintrans P, Bossert TJ, Sherry J, Kruk ME. A synthesis of implementation science frameworks and application to global health gaps. *Glob Health Res Policy.* 2019;4:25.

100. Gagnon MP, Nsangou É R, Payne-Gagnon J, Grenier S, Sicotte C. Barriers and facilitators to implementing electronic prescription: a systematic review of user groups' perceptions. *Journal of the American Medical Informatics Association : JAMIA.* 2014;21(3):535-541.

101. Harris M, Lawn SJ, Morello A, et al. Practice change in chronic conditions care: an appraisal of theories. *BMC health services research.* 2017;17(1):170.

102. Lennox L, Maher L, Reed J. Navigating the sustainability landscape: a systematic review of sustainability approaches in healthcare. *Implement Sci.* 2018;13(1):27.

103. Mitchell SA, Fisher CA, Hastings CE, Silverman LB, Wallen GR. A thematic analysis of theoretical models for translational science in nursing: mapping the field. *Nurs Outlook.* 2010;58(6):287-300.

104. Moullin JC, Sabater-Hernández D, Fernandez-Llimos F, Benrimoj SI. A systematic review of implementation frameworks of innovations in healthcare and resulting generic implementation framework. *Health Res Policy Syst.* 2015;13:16.

105. Nilsen P, Bernhardsson S. Context matters in implementation science: a scoping review of determinant frameworks that describe contextual determinants for implementation outcomes. *BMC health services research.* 2019;19(1):189.

106. Skolarus TA, Lehmann T, Tabak RG, Harris J, Lecy J, Sales AE. Assessing citation networks for dissemination and implementation research frameworks. *Implement Sci.* 2017;12(1):97.

107. Tabak RG, Khoong EC, Chambers DA, Brownson RC. Bridging research and practice: models for dissemination and implementation research. *Am J Prev Med.* 2012;43(3):337-350.

108. Wilson PM, Petticrew M, Calnan MW, Nazareth I. Disseminating research findings: what should researchers do? A systematic scoping review of conceptual frameworks. *Implement Sci.* 2010;5:91.

109. Wisdom JP, Chor KH, Hoagwood KE, Horwitz SM. Innovation adoption: a review of theories and constructs. *Adm Policy Ment Health.* 2014;41(4):480-502.

110. Aarons GA, Hurlburt M, Horwitz SM. Advancing a conceptual model of evidence-based practice implementation in public service sectors. *Adm Policy Ment Health.* 2011;38(1):4-23.

111. Allen LA, McIlvennan CK. Mis-GUIDED—The Importance of Negative Trials of Health Care Delivery and Implementation Science. *JAMA Cardiology.* 2020.

112. Andrews CM, D'Aunno TA, Pollack HA, Friedmann PD. Adoption of evidence-based clinical innovations: the case of buprenorphine use by opioid treatment programs. *Med Care Res Rev.* 2014;71(1):43-60.

113. Bayliss EA, Bonds DE, Boyd CM, et al. Understanding the context of health for persons with multiple chronic conditions: moving from what is the matter to what matters. *Ann Fam Med.* 2014;12(3):260-269.

114. Becker KD, Stirman SW. The science of training in evidence-based treatments in the context of implementation programs: current status and prospects for the future. *Adm Policy Ment Health.* 2011;38(4):217-222.

115. Beehler GP, Funderburk JS, Possemato K, Vair CL. Developing a measure of provider adherence to improve the implementation of behavioral health services in primary care: a Delphi study. *Implement Sci.* 2013;8:19.

116. Bradley KA, Ludman EJ, Chavez LJ, et al. Patient-centered primary care for adults at high risk for AUDs: the Choosing Healthier Drinking Options In primary CarE (CHOICE) trial. *Addict Sci Clin Pract.* 2017;12(1):15.

117. Broder-Fingert S, Walls M, Augustyn M, et al. A hybrid type I randomized effectiveness-implementation trial of patient navigation to improve access to services for children with autism spectrum disorder. *BMC Psychiatry.* 2018;18(1):79.

118. Cambon L, Minary L, Ridde V, Alla F. A tool to analyze the transferability of health promotion interventions. *BMC public health.* 2013;13:1184.

119. Candas B, Jobin G, Dubé C, et al. Barriers and facilitators to implementing continuous quality improvement programs in colonoscopy services: a mixed methods systematic review. *Endosc Int Open.* 2016;4(2):E118-133.

120. Chamberlain P, Brown CH, Saldana L. Observational measure of implementation progress in community based settings: the Stages of Implementation Completion (SIC). *Implement Sci.* 2011;6:116.

121. Chaudoir SR, Dugan AG, Barr CH. Measuring factors affecting implementation of health innovations: a systematic review of structural, organizational, provider, patient, and innovation level measures. *Implement Sci.* 2013;8:22.

122. Chor KH, Wisdom JP, Olin SC, Hoagwood KE, Horwitz SM. Measures for Predictors of Innovation Adoption. *Adm Policy Ment Health.* 2015;42(5):545-573.

123. Colldén C, Hellström A. Value-based healthcare translated: a complementary view of implementation. *BMC health services research.* 2018;18(1):681.

124. Damian AJ, Gallo J, Leaf P, Mendelson T. Organizational and provider level factors in implementation of trauma-informed care after a city-wide training: an explanatory mixed methods assessment. *BMC health services research.* 2017;17(1):750.

125. Faro JM, Orvek EA, Blok AC, et al. Dissemination and Effectiveness of the Peer Marketing and Messaging of a Web-Assisted Tobacco Intervention: Protocol for a Hybrid Effectiveness Trial. *JMIR Res Protoc.* 2019;8(7):e14814.

126. Fenwick KM, Palinkas LA, Hurlburt MS, Lengnick-Hall RD, Horwitz SM, Hoagwood KE. Acquisition of Information About Innovative Practices in Outpatient Mental Health Clinics. *Adm Policy Ment Health.* 2020:1-12.

127. Fitzgerald N, Platt L, Heywood S, McCambridge J. Large-scale implementation of alcohol brief interventions in new settings in Scotland: a qualitative interview study of a national programme. *BMC public health.* 2015;15:289.

128. Garland AF, Haine-Schlagel R, Brookman-Frazee L, Baker-Ericzen M, Trask E, Fawley-King K. Improving community-based mental health care for children: translating knowledge into action. *Adm Policy Ment Health.* 2013;40(1):6-22.

129. Gitlin LN, Marx K, Stanley IH, Hodgson N. Translating Evidence-Based Dementia Caregiving Interventions into Practice: State-of-the-Science and Next Steps. *Gerontologist.* 2015;55(2):210-226.

130. Goodman MS, Sanders Thompson VL. The science of stakeholder engagement in research: classification, implementation, and evaluation. *Translational behavioral medicine.* 2017;7(3):486-491.

131. Hartveit M, Hovlid E, Nordin MHA, et al. Measuring implementation: development of the implementation process assessment tool (IPAT). *BMC health services research.* 2019;19(1):721.

132. Herlitz L, MacIntyre H, Osborn T, Bonell C. The sustainability of public health interventions in schools: a systematic review. *Implement Sci.* 2020;15(1):4.

133. Hurlburt M, Aarons GA, Fettes D, Willging C, Gunderson L, Chaffin MJ. Interagency Collaborative Team Model for Capacity Building to Scale-Up Evidence-Based Practice. *Child Youth Serv Rev.* 2014;39:160-168.

134. Iliffe S, Manthorpe J, Drennan V, Goodman C, Warner J. The EVIDEM programme: a test for primary care research in London? *London J Prim Care (Abingdon).* 2008;1(2):69-73.

135. Johnson KE, Tachibana C, Coronado GD, et al. A guide to research partnerships for pragmatic clinical trials. *Bmj.* 2014;349:g6826.

136. King DK, Shoup JA, Raebel MA, et al. Planning for Implementation Success Using RE-AIM and CFIR Frameworks: A Qualitative Study. *Front Public Health.* 2020;8:59.

137. Kolko DJ, Hoagwood KE, Springgate B. Treatment research for children and youth exposed to traumatic events: moving beyond efficacy to amp up public health impact. *Gen Hosp Psychiatry.* 2010;32(5):465-476.

138. Kuiper D, Goedendorp MM, Sanderman R, Reijneveld SA, Steverink N. Identifying the determinants of use of the G&G interventions for older adults in health and social care: protocol of a multilevel approach. *BMC Res Notes.* 2015;8:296.

139. LaMonica HM, Davenport TA, Braunstein K, et al. Technology-Enabled Person-Centered Mental Health Services Reform: Strategy for Implementation Science. *JMIR Ment Health.* 2019;6(9):e14719.

140. Leeman J, Baquero B, Bender M, et al. Advancing the use of organization theory in implementation science. *Prev Med.* 2019;129s:105832.

141. Lengnick-Hall R, Willging C, Hurlburt M, Fenwick K, Aarons GA. Contracting as a bridging factor linking outer and inner contexts during EBP implementation and sustainment: a prospective study across multiple U.S. public sector service systems. *Implement Sci.* 2020;15(1):43.

142. Lennox L, Doyle C, Reed JE, Bell D. What makes a sustainability tool valuable, practical and useful in real-world healthcare practice? A mixed-methods study on the development of the Long Term Success Tool in Northwest London. *BMJ Open.* 2017;7(9):e014417.

143. Lewis CC, Stanick CF, Martinez RG, et al. The Society for Implementation Research Collaboration Instrument Review Project: a methodology to promote rigorous evaluation. *Implement Sci.* 2015;10:2.

144. Lloyd HM, Ekman I, Rogers HL, et al. Supporting Innovative Person-Centred Care in Financially Constrained Environments: The WE CARE Exploratory Health Laboratory Evaluation Strategy. *International journal of environmental research and public health.* 2020;17(9).

145. Martinez RG, Lewis CC, Weiner BJ. Instrumentation issues in implementation science. *Implement Sci.* 2014;9:118.

146. McAlearney AS, Walker DM, Livaudais-Toman J, Parides M, Bickell NA. Challenges of implementation and implementation research: Learning from an intervention study designed to improve tumor registry reporting. *SAGE Open Med.* 2016;4:2050312116666215.

147. McCullough MB, Chou AF, Solomon JL, et al. The interplay of contextual elements in implementation: an ethnographic case study. *BMC health services research.* 2015;15:62.

148. Morrato EH, Rabin B, Proctor J, et al. Bringing it home: expanding the local reach of dissemination and implementation training via a university-based workshop. *Implement Sci.* 2015;10:94.

149. Munoz-Plaza CE, Parry C, Hahn EE, et al. Integrating qualitative research methods into care improvement efforts within a learning health system: addressing antibiotic overuse. *Health Res Policy Syst.* 2016;14(1):63.

150. Nadeem E, Jaycox LH, Kataoka SH, Langley AK, Stein BD. Going to Scale: Experiences Implementing a School-Based Trauma Intervention. *School Psych Rev.* 2011;40(4):549-568.

151. Nadeem E, Gleacher A, Beidas RS. Consultation as an implementation strategy for evidence-based practices across multiple contexts: unpacking the black box. *Adm Policy Ment Health.* 2013;40(6):439-450.

152. Nadeem E, Gleacher A, Pimentel S, Hill LC, McHugh M, Hoagwood KE. The role of consultation calls for clinic supervisors in supporting large-scale dissemination of evidence-based treatments for children. *Adm Policy Ment Health.* 2013;40(6):530-540.

153. Nadeem E, Olin SS, Hill LC, Hoagwood KE, Horwitz SM. Understanding the components of quality improvement collaboratives: a systematic literature review. *Milbank Q.* 2013;91(2):354-394.

154. Nadeem E, Olin SS, Hill LC, Hoagwood KE, Horwitz SM. A literature review of learning collaboratives in mental health care: used but untested. *Psychiatr Serv.* 2014;65(9):1088-1099.

155. O'Malley G, Asrat L, Sharma A, et al. Nurse task shifting for antiretroviral treatment services in Namibia: implementation research to move evidence into action. *PLoS One.* 2014;9(3):e92014.

156. Okere NE, Urlings L, Naniche D, de Wit TFR, Gomez GB, Hermans S. Evaluating the sustainability of differentiated service delivery interventions for stable ART clients in sub-Saharan Africa: a systematic review protocol. *BMJ Open.* 2020;10(1):e033156.

157. Olin SC, Chor KH, Weaver J, et al. Multilevel predictors of clinic adoption of state-supported trainings in children's services. *Psychiatr Serv.* 2015;66(5):484-490.

158. Olin SS, Hemmelgarn AL, Madenwald K, Hoagwood KE. An ARC-Informed Family Centered Care Intervention for Children's Community Based Mental Health Programs. *J Child Fam Stud.* 2016;25(1):275-289.

159. Pannebakker NM, Fleuren MAH, Vlasblom E, Numans ME, Reijneveld SA, Kocken PL. Determinants of adherence to wrap-around care in child and family services. *BMC health services research.* 2019;19(1):76.

160. Pednekar MS, Nagler EM, Gupta PC, et al. Scaling up a tobacco control intervention in low resource settings: a case example for school teachers in India. *Health Educ Res.* 2018;33(3):218-231.

161. Pescheny JV, Pappas Y, Randhawa G. Evaluating the Implementation and Delivery of a Social Prescribing Intervention: A Research Protocol. *Int J Integr Care.* 2018;18(1):13.

162. Prendergast M, Welsh WN, Stein L, et al. Influence of Organizational Characteristics on Success in Implementing Process Improvement Goals in Correctional Treatment Settings. *J Behav Health Serv Res.* 2017;44(4):625-646.

163. Proctor EK, Powell BJ, Baumann AA, Hamilton AM, Santens RL. Writing implementation research grant proposals: ten key ingredients. *Implement Sci.* 2012;7:96.

164. Ramsey AT, Proctor EK, Chambers DA, et al. Designing for Accelerated Translation (DART) of Emerging Innovations in Health. *J Clin Transl Sci.* 2019;3(2-3):53-58.

165. Ratcliff CG, Vinson CA, Milbury K, Badr H. Moving family interventions into the real world: What matters to oncology stakeholders? *J Psychosoc Oncol.* 2019;37(2):264-284.

166. Robins LS, Jackson JE, Green BB, Korngiebel D, Force RW, Baldwin LM. Barriers and facilitators to evidence-based blood pressure control in community practice. *J Am Board Fam Med.* 2013;26(5):539-557.

167. Rongey C, Asch S, Knight SJ. Access to care for vulnerable veterans with hepatitis C: a hybrid conceptual framework and a case study to guide translation. *Translational behavioral medicine.* 2011;1(4):644-651.

168. Rotteau L, Webster F, Salkeld E, et al. Ontario's emergency department process improvement program: the experience of implementation. *Acad Emerg Med.* 2015;22(6):720-729.

169. Stirman SW, Pontoski K, Creed T, et al. A Non-randomized Comparison of Strategies for Consultation in a Community-Academic Training Program to Implement an Evidence-Based Psychotherapy. *Adm Policy Ment Health.* 2017;44(1):55-66.

170. Wiltsey Stirman S, Marques L, Creed TA, et al. Leveraging routine clinical materials and mobile technology to assess CBT fidelity: the Innovative Methods to Assess Psychotherapy Practices (imAPP) study. *Implement Sci.* 2018;13(1):69.

171. Sullivan JL, Adjognon OL, Engle RL, et al. Identifying and overcoming implementation challenges: Experience of 59 noninstitutional long-term services and support pilot programs in the Veterans Health Administration. *Health Care Manage Rev.* 2018;43(3):193-205.

172. Sullivan JL, Engle RL, Tyler D, et al. Is Variation in Resident-Centered Care and Quality Performance Related to Health System Factors in Veterans Health Administration Nursing Homes? *Inquiry.* 2018;55:46958018787031.

173. Swinkels ICS, Huygens MWJ, Schoenmakers TM, et al. Lessons Learned From a Living Lab on the Broad Adoption of eHealth in Primary Health Care. *J Med Internet Res.* 2018;20(3):e83.

174. Teri L, McKenzie G, Logsdon RG, et al. Translation of two evidence-based programs for training families to improve care of persons with dementia. *Gerontologist.* 2012;52(4):452-459.

175. Trompette J, Kivits J, Minary L, Cambon L, Alla F. Stakeholders' perceptions of transferability criteria for health promotion interventions: a case study. *BMC public health.* 2014;14:1134.

176. Urquhart R, Kendell C, Geldenhuys L, et al. The role of scientific evidence in decisions to adopt complex innovations in cancer care settings: a multiple case study in Nova Scotia, Canada. *Implement Sci.* 2019;14(1):14.

177. Van Acker R, De Bourdeaudhuij I, De Cocker K, Klesges LM, Willem A, Cardon G. Sustainability of the whole-community project '10,000 Steps': a longitudinal study. *BMC public health.* 2012;12:155.

178. Ward MM, Baloh J, Zhu X, Stewart GL. Promoting Action on Research Implementation in Health Services framework applied to TeamSTEPPS implementation in small rural hospitals. *Health Care Manage Rev.* 2017;42(1):2-13.

179. Weaver NL, Kortlandt V, Williams J, et al. Assessing Community-Based Injury Prevention Services in U.S. Children's Hospitals. *AIMS Public Health.* 2014;1(4):199-210.

180. Weaver NL, Buskirk TD, Jupka K, Williams J. Organizational factors related to the adoption of an injury prevention program by U.S. children's hospitals. *Translational behavioral medicine.* 2019;9(4):768-776.

181. Welsh WN, Knudsen HK, Knight K, et al. Effects of an Organizational Linkage Intervention on Inter-Organizational Service Coordination Between Probation/Parole Agencies and Community Treatment Providers. *Adm Policy Ment Health.* 2016;43(1):105-121.
